# Quantifying the role of weather on seasonal influenza

**DOI:** 10.1186/s12889-016-3114-x

**Published:** 2016-05-26

**Authors:** Marion Roussel, Dominique Pontier, Jean-Marie Cohen, Bruno Lina, David Fouchet

**Affiliations:** University Lyon 1, CNRS, UMR 5558, Biometry and Evolutionary Biology laboratory, Bât. Grégor Mendel 43 bd du 11 novembre 1918, Villeurbanne Cedex, F-69622 France; LabEx ECOFECT, Eco-evolutionary Dynamics of infectious Diseases, University of Lyon, Lyon, France; OPEN ROME, Paris, France; Laboratory of Virology, Centre National de Référence des Virus Influenzae, Hospices Civils de Lyon, Lyon, France; Virpath, EA4610, Faculty of Medecine Lyon Est, University Claude Bernard Lyon 1, Cedex08, Lyon, 69372 France

## Abstract

**Background:**

Improving knowledge about influenza transmission is crucial to upgrade surveillance network and to develop accurate predicting models to enhance public health intervention strategies. Epidemics usually occur in winter in temperate countries and during the rainy season for tropical countries, suggesting a climate impact on influenza spread. Despite a lot of studies, the role of weather on influenza spread is not yet fully understood. In the present study, we investigated this issue at two different levels.

**Methods:**

First, we evaluated how weekly (intra-annual) incidence variations of clinical diseases could be linked to those of climatic factors. We considered that only a fraction of the human population is susceptible at the beginning of a year due to immunity acquired from previous years. Second, we focused on epidemic sizes (cumulated number of clinical reported cases) and looked at how their inter-annual and regional variations could be related to differences in the winter climatic conditions of the epidemic years over the regions. We quantified the impact of fifteen climatic variables in France using the Réseau des GROG surveillance network incidence data over eleven regions and nine years.

**Results:**

At the epidemic scale, no impact of climatic factors was highlighted. At the intra-annual scale, six climatic variables had a significant impact: average temperature (5.54 ± 1.09 %), absolute humidity (5.94 ± 1.08 %), daily variation of absolute humidity (3.02 ± 1.17 %), sunshine duration (3.46 ± 1.06 %), relative humidity (4.92 ± 1.20 %) and daily variation of relative humidity (4.46 ± 1.24 %). Since in practice the impact of two highly correlated variables is very hard to disentangle, we performed a principal component analysis that revealed two groups of three highly correlated climatic variables: one including the first three highlighted climatic variables on the one hand, the other including the last three ones on the other hand.

**Conclusions:**

These results suggest that, among the six factors that appeared to be significant, only two (one per group) could in fact have a real effect on influenza spread, although it is not possible to determine which one based on a purely statistical argument. Our results support the idea of an important role of climate on the spread of influenza.

**Electronic supplementary material:**

The online version of this article (doi:10.1186/s12889-016-3114-x) contains supplementary material, which is available to authorized users.

## Background

Influenza is one of the most significant diseases in humans, generating worldwide annual epidemics, which result in about three to five million cases of severe illness, and about 250,000 to 500,000 deaths [1]. Improving influenza knowledge about key epidemiological parameters such as survival, transmission and reproduction in hosts is essential to upgrade surveillance network and to develop more accurate predicting models. Better epidemic predictions would set up more appropriate public health prevention and intervention strategies.

Epidemics occur mainly during the winter season months in temperate countries [2–4] unlike in tropical and sub-tropical countries where they generally happen during the rainy season [5–8]. These differences suggest a climate impact on influenza spread. Climate might affect influenza diffusion (onset, duration, size) by impacting individuals’ contact rates (frequency and duration), population immunity and virus survival outside human body. The role of weather is however not fully understood [9] despite a lot of laboratory studies of host susceptibility according to environmental conditions [10–12] and mathematical modeling approaches analyzing the link between influenza morbidity or mortality and climatic factors [13–18].

Various climatic factors such as temperature, humidity, rainfalls, UV radiation, sunshine duration and wind speed might have an impact on influenza spread. In temperate countries, humidity and temperature might play an important role in influenza spread. Several laboratory works showed that a cold and dry weather promotes a higher virus survival outside human body and a better transmission [11, 19]. Cold air inhalation chills nasal epithelium leading to an inhibition of mechanical defenses of the respiratory mucosa and of the immune system [20]. Otherwise models explaining influenza epidemics (e.g., onset, peak, mortality) according to climatic factors reinforce the role of humidity and temperature in influenza spread in the United States [13, 15] as well as in Europe [16, 21]. Rainfalls might have an impact in tropical and sub-tropical countries such as in Central and South America [22–24] and in Asia [25, 26]. Another theory suggests a link between vitamin D secretion and influenza immunity, which is supported by experiments [27, 28]. As UV radiation is involved in vitamin D production, a lack of UV radiation in winter, for temperate countries, leads to a reduction of vitamin D production and might boost influenza epidemics [29, 30]. Dowell [31] also suggested a role of dark/light cycles and photoperiod on the immune systems caused by melatonin fluctuations. Thereby UV radiation and sunshine duration might have an indirect effect on influenza infections. Finally in China, Xiao et al. [32] proposed that a low wind speed contribute to influenza spread. In fact a strong wind speed may have a dispersive effect on influenza in the environment limiting its diffusion.

The aim of this study is to quantify the impact of several climatic factors such as temperature, humidity, and rainfalls, on influenza epidemics in France, a temperate country. The role of weather can be estimated based on the variation of influenza propagation in an area according to its climate variation. Usually studies compared observed to modeled epidemics taking into account climatic factors by comparing incidence or mortality within an epidemic year [13–18]. The impact of the climatic factors included in the model is supported if modeled and observed epidemics are similar. However little information is available about influenza transmission. Modeling approaches made a lot of hypotheses about the within host virus dynamic such as incubation and infectious periods *R*_0_ etc. Such hypotheses may have a strong impact on influenza propagation, which might lead to a misestimating of climatic effects. In order to reduce the set of model hypotheses, we built an autoregressive model based on the shape of the observed epidemics over time. We explained the intraseasonal variation of incidence of eleven French regions and for nine epidemic years (an epidemic year corresponds to October of a year until April of the year after) with the climatic variables listed before, to quantify their respective impact globally over all regions, then specifically in each region for significant climatic variables. The originality of our model is to consider that only a fraction of the human population is susceptible at the beginning of a year due to immunity acquired from previous years. Considering loss of immunity in modeling influenza epidemics might be important [33] even if almost no studies about influenza and climate take it into account to our knowledge. Here we called susceptible individuals people that could be infected and develop symptoms, as we only had data about infected people presenting symptoms. We then quantified potential effects of climatic factors on the interseasonal variation of influenza epidemics. To do that we built an autoregressive linear model that explains the epidemic size according to the average value of the climatic factors over an epidemic year for the nine epidemic years and the eleven French regions.

## Methods

### Data

#### Epidemiological data

Epidemiological data come from the Réseau des GROG (Regional Influenza Surveillance Group) sentinel network, which is a French surveillance network made up of general practitioners and pediatricians. These physician sentinels identify cases of respiratory pathogens including influenza. Each region has on average 25 sentinels (from 10 to 75 depending on regions and epidemic years) involved in the Réseau des GROG sentinel network. Every week from October to April, they describe in reports the intensity of their activity by giving the number of days they worked, the number of medical acts performed and the number of acute respiratory infection (ARI) defined as the sudden onset of at least one respiratory sign (cough, rhinitis, coryza, etc.) and at least one systemic sign suggesting an acute infectious context (fever, fatigue, headache, myalgia, malaise, etc.). In addition, sentinels randomly realize nasal/pharyngeal swab samples on patients with a less than 48 h ARI. Analysis of these samples allows virological confirmation of influenza infections. Using the weekly information reported by each physician sentinel (clinical reports and virological samples analysis), the Réseau des GROG sentinel network is able to provide an estimate of the number of influenza-infected individuals called the influenza incidence.

First they define the ARI incidence, the number of ARI cases (*I*_*ARI*_), for a region and a week *t* as:

$$ {I}_{ARI}(t)=\left(\frac{Pe{d}_{region}}{Pe{d}_{GROG\; participants}(t)}\cdot AR{I}_{Ped}(t)\right)+\left(\frac{G{P}_{region}}{G{P}_{GROG\; participants}(t)}\cdot AR{I}_{GP}(t)\right) $$

where *ARI*_*GP*_(*t*) and *ARI*_*Ped*_(*t*) stand for the number of ARI cases for week *t*, respectively, reported by general practitioners (GP) and pediatricians from the Réseau des GROG sentinel network. *GP*_*region*_ and *Ped*_*region*_ are, respectively, the number of GP and pediatricians of a region. *GP*_*GROG participants*_(*t*) and *Ped*_*GROG participants*_(*t*) represent the number of GP and pediatricians who participated in surveillance the week *t*, respectively. Age of infected individuals was not taken into account assuming that climatic factors have a uniform impact on influenza spread within the population.

Second, the Réseau des GROG sentinel network estimates influenza incidence relying on both the ARI incidence and virological data. For each week of each region, an influenza positivity rate (for all circulating strains) is defined as the ratio of the number of positive samples on the total number of samples collected over a week. It is calculated using a moving average of order 3 taking into account the positive rate of the week concerned and the ones before and after in order to remove excessive fluctuations. We assumed that the positive rate corresponds to the actual proportion of influenza cases among ARI cases reported by the Réseau des GROG sentinel network. The influenza incidence (*I*_*influenza*_) is defined as the ARI incidence weighted by the positivity rate (*T*_+_):

$$ {I}_{influenza}(t)={I}_{ARI}(t) \cdot {T}_{+}(t) $$

Epidemiological data are available from the 2003–2004 epidemic year to the 2012–2013 epidemic year. However we excluded the 2009–2010 epidemic year where the H1N1 pandemic happened in order to study only seasonal epidemics.

#### Climatic data

**Fig. 1:**
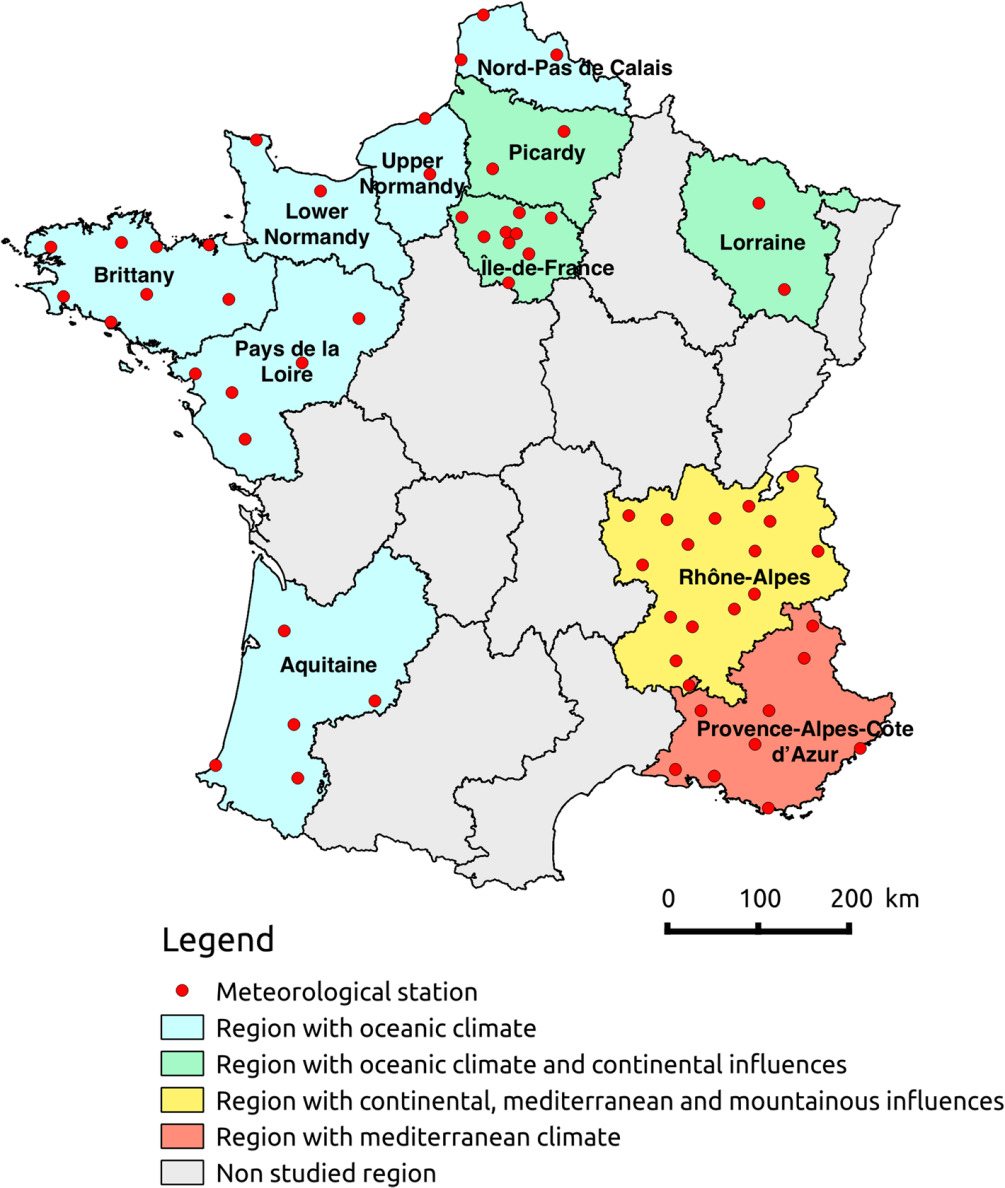
Map of France showing the eleven studied regions according to their climate: Aquitaine, Lower Normandy, Brittany, Upper Normandy, Nord-Pas de Calais and Pays de la Loire in *blue* for their oceanic climate, Île-de-France, Lorraine, and Picardy in *green* for their oceanic climate with continental influences, PACA in *orange* for its Mediterranean climate and Rhône-Alpes in *yellow* for its continental, Mediterranean and mountainous influences, with the geographical location of the 65 meteorological stations (*in red*)

We chose eleven French regions: Aquitaine, Lower Normandy, Brittany, Upper Normandy, Île-de-France, Lorraine, Nord-Pas-de-Calais, Pays de la Loire, Picardy, Provence-Alpes-Côte d’Azur (PACA) and Rhône-Alpes, which have different climates. Aquitaine, Pays de la Loire, Brittany, Lower Normandy, Upper Normandy and Nord-Pas de Calais have an oceanic climate; Île-de-France, Picardy and Lorraine have an oceanic climate with continental influences; PACA has a Mediterranean climate and Rhône-Alpes climate is made up of continental, Mediterranean and mountainous influences (see Fig. 1).

Climatic data were provided by Météo-France, the French national meteorological service. We picked 65 meteorological stations (see Fig. 1) to collect data in order to estimate climatic factors that globally describe each region. We had information on temperature, relative humidity, absolute humidity, rainfalls, sunshine duration (very correlated to UV radiation), and wind speed (see Additional file 1). It is not necessarily easy to choose efficient climatic factors, as illustrated by Davis et al. [34] who highlighted the challenge of selecting an appropriate measure of the humidity covariate.

As epidemiological data were weekly available, we created weekly climatic variables from the daily meteorological data by averaging the daily data. The climatic variables built are defined in Table 1.

**Table 1:** Definition of the climatic variables

Climatic variable	Formula
Average temperature	$\frac{1}{days}\cdot \frac{1}{stations}\cdot {\displaystyle {\sum}_{j=0}^{days}{\displaystyle {\sum}_{s=0}^{stations} average\ daily\ temperature\left(j,s\right)}}$
Daily variation of temperature	$\frac{1}{days}\cdot \frac{1}{stations}\cdot {\displaystyle {\sum}_{j=0}^{days}{\displaystyle {\sum}_{s=0}^{stations}\left( \max daily\ temperature\left(j,s\right)- \min daily temperature\left(j,s\right)\right)}}$
Relative weekly variation of temperature	*Average temperature*(*week*_*t* + 1_) − *Average temperature*(*week*_*t*_)
Absolute weekly variation of temperature	|*Average temperature*(*week*_*t* + 1_) − *Average temperature*(*week*_*t*_)|
Average relative humidity	$\frac{1}{days}\cdot \frac{1}{stations}\cdot {\displaystyle {\sum}_{j=0}^{days}{\displaystyle {\sum}_{s=0}^{stations} average\ daily\ relative\ humidity\left(j,s\right)}}$
Daily variation of relative humidity	$\frac{1}{days}\cdot \frac{1}{stations}\cdot {\displaystyle {\sum}_{j=0}^{days}{\displaystyle {\sum}_{s=0}^{stations}\left( \max daily\ relative\ humidity\left(j,s\right)- \min daily\ relative\ humidity\left(j,s\right)\right)}}$
Relative weekly variation of relative humidity	*Average relative humidity*(*week*_*t* + 1_) − *Average relative humidity*(*week*_*t*_)
Absolute weekly variation of relative humidity	|*Average relative humidity*(*week*_*t* + 1_) − *Average relative humidity*(*week*_*t*_)|
Average absolute humidity	$\frac{1}{days}\cdot \frac{1}{stations}\cdot {\displaystyle {\sum}_{j=0}^{days}{\displaystyle {\sum}_{s=0}^{stations} average\ daily\ absolute\ humidity\left(j,s\right)}}$
Daily variation of absolute humidity	$\frac{1}{days}\cdot \frac{1}{stations}\cdot {\displaystyle {\sum}_{j=0}^{days}{\displaystyle {\sum}_{s=0}^{stations}\left( \max daily\ absolute\ humidity\left(j,s\right)- \min daily\ absolute\ humidity\left(j,s\right)\right)}}$
Relative weekly variation of absolute humidity	*Average absolute humidity*(*week*_*t* + 1_) − *Average absolute humidity*(*week*_*t*_)
Absolute weekly variation of absolute humidity	|*Average absolute humidity*(*week*_*t* + 1_) − *Average absolute humidity*(*week*_*t*_)|
Average wing speed	$\frac{1}{days}\cdot \frac{1}{stations}\cdot {\displaystyle {\sum}_{j=0}^{days}{\displaystyle {\sum}_{s=0}^{stations} average\ daily\ wind\ speed\left(j,s\right)}}$
Rainfall height	$\frac{1}{days}\cdot \frac{1}{stations}\cdot {\displaystyle {\sum}_{j=0}^{days} average\ daily\ rainfalls\ height\left(j,s\right)}$
Sunshine duration	$\frac{1}{days}\cdot \frac{1}{stations}\cdot {\displaystyle {\sum}_{j=0}^{days} average\ daily\ sunshine\ duration\left(j,s\right)}$

### Mathematical models

Climatic factors can impact influenza spread by both increasing the transmissibility of the virus and/or by increasing the susceptibility of its human host. One particularity of our data set is that the variability in influenza incidence is reported at different scales: the transmission scale (intraseasonal variation) and the epidemic scale (interseasonal variation). The impact of climatic factors may occur at the two scales in which it will be observed in a slightly different way.

At the transmission scale – during a seasonal epidemic of a given year in a given region – favorable climatic (for influenza diffusion) factors will lead to observe an increase in disease (apparent) transmission. At this scale we will search for significant associations between weekly variations of climatic factors and those of the disease apparent transmission rate (defined below). Different observed epidemics (in all regions and epidemic years) will be treated as independent replicates.

At the epidemic scale - the impact of a climatic factor (in a region over an entire epidemic year) may mainly be observed by the increase or decrease in the epidemic size (the total number of infected individuals). At this scale we will search for significant associations between the size of the epidemic and the average value of the different climatic factors (over an epidemic year in a region). Because both scales imply different response variables, they will be treated separately and independently.

#### Impact of climatic factors at the transmission scale

We built an auto-regressive statistical model with a lag of one week to explain variations in the weekly influenza incidence with climatic factors for eleven French regions over nine epidemic years.

Our model is inspired from general epidemiological models in which the number of infected and symptomatic individuals at time *t*, *I*(*t*), is modeled as a general function depending on i) the number of infected and symptomatic at time *t* − 1, *I*(*t* − 1), and ii) the number of individuals at time *t* who are susceptible to develop the symptomatic form of the disease upon infection, *S*(*t* − 1):

1 $$ I(t) = \beta (t)\cdot I{\left(t-1\right)}^a\cdot S{\left(t-1\right)}^b $$

where a and b are constants (heterogeneity parameters) extending the mass action type model into a more general form, which has been shown as a relevant way to approximate epidemic shapes in populations with heterogeneous mixing [35]. *β* is the apparent transmission rate of the virus. *a* = *b* = 1 correspond to the mass action model [36]. With a logarithm transformation the relationship becomes:

2 $$ \log \left(I(t)\right)= \log \left(\beta (t)\right)+a\cdot \log \left(I\left(t-1\right)\right) + b\cdot log\left(S\left(t-1\right)\right) $$

In fact, the numbers of infected and susceptible individuals are not directly observed. *Î* and *Ŝ* denote estimates of the number of infected and susceptible individuals, respectively. Considering that i) the number infected and susceptible individuals are estimated and ii) there is stochasticity in the transmission process, the relationship (2) becomes:

3 $$ \log \left(\widehat{I}(t)\right)= \log \left(\beta (t)\right)+a\cdot \log \left(\widehat{I}\left(t-1\right)\right) + b\cdot \log \left(\widehat{S}\left(t-1\right)\right)+{\varepsilon}_1 $$

To analyze the impact of a climatic factor (*F*_*c*_), we considered that the transmission rate is given by:

4 $$ \log \left(\beta (t)\right) = c\cdot {F}_c+d+{\varepsilon}_2 $$

where *c* quantifies the link between *F*_*c*_ and *β*, *d* is a constant and *ε*_2_ is a random term independent of *F*_*c*_ modeling the fluctuation in *β* independent of *F*_*c*_, i.e., due to other factors.

Not all the human population is susceptible to influenza, e.g., due to immunity acquired from previous infection. However, giving an estimate of the influenza susceptible population (non-immune population) is difficult due to the seasonal variation of circulating strains, loss of immunity phenomena and the fact that asymptomatic cases are not detected. In this model, we keep a pragmatic statistical view by considering that the susceptible pool linearly decreases every week with the infection of new individuals. So the estimated susceptible population *Ŝ* for a week *t* and a given region is given by:

5 $$ \widehat{S}(t) = \widehat{N}-{\widehat{I}}_{cum}\left(t-1\right) $$

where *Î*_*cum*_ is the number of infected individuals cumulated from the beginning of the epidemic year to the week *t* − 1. Note that introducing *Î*_*cum*_(*t* − 1) in the model implicitly introduces a link between *I*(*t*) and *I*(*t* − 2), *I*(*t* − 3), etc. in our model. $\widehat{\mathrm{N}}$ is a statistical (constant in time) parameter introduced to model a linear relationship between the number of individuals that are susceptible to develop the symptomatic form of the influenza infection and the cumulated number of individuals that developed a symptomatic influenza infection until *t* − 1. On a biological point of view, it can be interpreted as the total number of individuals that could potentially develop an observable form of the disease upon infection, but this interpretation has to be taken with caution (see Discussion). Combining equations (3), (4) and (5) we get:

6 $$ Y(t) = c\cdot {F}_c + d+a\cdot Y\left(t-1\right)+b\cdot \log \left(\widehat{N}-{\widehat{I}}_{cum}\left(t-1\right)\right)+\varepsilon $$

where *Y*(*t*) is the logarithm of the estimated number of infected individuals. *ε* = *ε*_1_ + *ε*_2_ is the total residual error and it is assumed to be distributed according to a Gaussian centered distribution with a standard deviation *σ*.

We defined $\widehat{\alpha}=\frac{I_{Tmax}}{\widehat{N}}$, which provides an estimate of the proportion of individuals who developed the disease (with symptoms) in the pool of individuals that could have developed it. *I*_*Tmax*_ denotes here the time at which the influenza surveillance ends (mid-April). *α* = 1 means that all individuals who could potentially become sick acquired the infection, and suggests that the disease has a sufficient transmission to reach the entire susceptible pool of the population. At the opposite *α* < 1 suggests that the virus spread was not sufficient to reach the entire susceptible pool.

Since all the model coefficients (*a*, *b*, *c*, *d* and *α*) may depend on both the region (*R*) and the epidemic year (*Y*), there are many possible different models that can be considered depending on how *Y* and *R* affect the coefficients. Models are synthesized as follows:

$$ a(X),\ b(Z),\ c(U),\ d(V),\ \alpha (W) $$

where *X*, *Z*, *U*, *V* and *W* are formulas depending on *R* and *Y*. To take a few examples, be *x* a generic variable that can be *a*, *b*, *c*… *x*(0) means that *x* = 0 in the model; *x*(*1*) means that *x* is constant (intercept model); *x*(*R*) means that *x* depends on the region; *x*(*R* + *Y*) means that *x* depends on both the region and epidemic year in an additive way and *x*(*R* ⋅ *Y*) in a multiplicative way.

The most complicated model considered (i.e., the complete model) is not the model where all parameters depend multiplicatively on *R* and *Y* (*R* ⋅ *Y*), which would contain too many parameters to be tractable. Since *a* and *b* are shape parameters for the spread of the epidemic, it is reasonable to assume that they are characteristics of the region (*a*(*R*) and *b*(*R*)). *d* affects the average transmission rate of the virus. It can be different between regions (which show different demographic characteristics) and between epidemic years (because the circulating influenza strain is different from one epidemic year to the next), but it is reasonable to consider that it will only be slightly affected by the interaction between these two factors (*d*(*R* ⋅ *Y*)). That is why the most complicated model considered was *a*(*R*), *b*(*R*), *c*(*R*), *d*(*R* + *Y*), *α*(*R* ⋅ *Y*).

Model parameters were inferred using maximum likelihood estimation. The analysis was performed following two steps. In the first step, we tried to reduce as much as possible the complexity of the model that will be used to test climatic factors and estimate their impact. An AIC criterion was used to select the model having the lowest AIC. If the difference between two AIC values is less than two, the most parsimonious model is chosen. In that procedure, the coefficient *c* was fixed to zero (model *c*(0)) in order to select a model that is independent of climatic data. In the second step, climatic factors were introduced in the model selected in step 1. In this section, we search how increases or decreases in the value of climatic factors during an epidemic can impact the apparent transmission rate. Global variations in the average value of the climatic factors between regions and epidemic years are not interesting here. That is why climatic factors were first centered within years and regions: for a climatic factor *f* measured during a week *t*, an epidemic year *Y* and a region *R*, we define:

$$ {\varphi}_{t,Y,R} = {f}_{t,Y,R} - \overline{f_{Y,R}} $$

where $\overline{f_{Y,R}}$ denotes the mean of climatic factor *f* over the surveillance period of epidemic year *Y* in region *R*. To allow easy comparison between the estimated coefficients of the fifteen climatic factors, each of them was then reduced:

$$ {F}_{t,Y,R}=\frac{\varphi_{t,Y,R}}{s{d}_{\varphi }} $$

where *sd*_*φ*_ stands for the standard deviation of the variable *φ*. over all epidemic weeks *t*, epidemic year *Y* and region *R*.

In total, fifteen climatic factors were tested, leading to potentially important problems of multiple testing. Since climatic factors are not independent, applying a simple Bonferroni correction would lead to a loss of statistical power [37]. Instead, we preferred a multiple testing correction based on permutation tests [38]. The idea of the permutation test we developed here is to keep the same values for all the climatic factors but to shuffle the week indexes, within a given region and a given epidemic year (in order to break the potential association between any climatic factor and the observed course of the epidemic). Mathematically, let us call *F*_*t*,*Y*,*R*_ the value of the climatic factor *F* during the *t*^*th*^ week of region *R* and epidemic year *Y*. Let us call *P* a permutation of the week indexes *t*. The permuted climatic factors (*F*) associated to permutation *P* in region *R* and year *Y* will be defined by: *F*_*P*(*t*),*Y*,*R*_. The main advantage of this permutation procedure is that it conserves the within epidemic year and region correlation structure between the climatic factors. One permutation of the climatic factors is then defined as a set of permutations (one for each epidemic year in each region) leading to a set of permuted climatic factors in all regions and for all epidemic years. Note that these permuted factors have strictly no reason to be correlated with the apparent disease transmission rate (the permutation is purely random) and hence can be considered as realizations of the null hypothesis H0 “the apparent transmission rate is not linked to any climatic factor”.

We used the absolute value of the maximum estimated climatic factor coefficients (*c*_*max*_) as a test statistic for H0. We generated 10,000 permutations of climatic factors (see above) and for each one we calculated *c*_*max*_, leading to 10,000 independent realizations of *c*_*max*_ under H0. The 95 % quantile of the distribution defines a significant threshold. Climatic factors are considered being significantly linked to the apparent transmission rate if the absolute value of their *c* estimate from data is above the defined threshold. Model parameters are estimated using maximum likelihood. Standard errors of the estimations of the model parameters were determined using the square roots of the diagonal elements of the covariance matrix (the inverse of the negative of the expected value of the Hessian matrix). Model implementation and permutation tests were performed in Python.

#### Impact of climatic factors at the epidemic scale

To evaluate the impact of climatic factors at the epidemic scale we considered the ratio of cumulated number of infected individuals across the entire epidemic period (from the first week of epidemic of the first region in epidemic to the last week of epidemic of the last region in epidemic) to the total population – an indicator of the epidemic size – as a response variable (*ES*).

As individuals infected a previous year are immunized the year after if there is not much influenza virus evolution (i.e., antigenic drifts), the epidemic size of a previous year determines the number of susceptible individuals the year after. We expected a negative correlation between the epidemic size of a previous year and the one the year after, because if the epidemic size was high on the previous year, there will be less susceptible individuals the year after, leading to a smaller epidemic. That is why we considered an autoregressive linear model in order to take into account the correlation between the epidemic size of an epidemic year and the one from the previous epidemic year. We used a logarithm transformation in order to fit the normality and the homoscedasticity of residuals. The model is defined as:

$$ \log \left(E{S}_{Y,R}\right) = {a}_0+{a}_Y+{a}_R+b\cdot \log \left(E{S}_{Y-1,R}\right)+c\cdot \overline{F_{Y,R}} $$

where *a*_0_, *b* and *c* are constant model parameters and *a*_*Y*_ (respectively *a*_*R*_) models potential systematic variations in the epidemic size between epidemic years (respectively regions). These two terms account for the fact that some regions may be more prone to important epidemics (e.g., due to population demography) and the strains circulating some epidemic years can be more virulent or affect a larger set of the human population due to more important genetic differences with the strains of the previous epidemic years. $\overline{F_{Y,R}}$ denotes the mean value of climatic factor *F* over the entire epidemic year.

Foremost we selected model parameters (*a*_*Y*_, *a*_*R*_ and *b*) using an AIC criterion and then we assessed the impact of climatic factors.

Multiple hypothesis testing was corrected as in the previous section. Values of *Y* and *R* were shuffled together (pairs of values for *Y* and *R* were randomly re-attributed to all epidemics). For a permutation *P*, new climatic factors were built as $\overline{F_{P\left(Y,R\right)}}$. The advantage of this permutation procedure is that, as above, it keeps the covariance structure between the climatic factors. As previously the permutation test is used to determine a significant threshold for the *c* coefficients using the maximum absolute estimated value of the *c* coefficients as a statistic.

Model parameters were estimated using the classical tools of linear models implemented in R3.1.2 [39].

## Results

### Impact of climatic factors at the transmission scale

In order to reduce the complexity of the model we performed an AIC selection without climatic factors. According to the AIC criterion we chose the model with all coefficients (*a*, *b*, *d* and *α*) independent of regions and epidemic years (see Table 2). Then we built models adding each climatic factor to the chosen model. Finally we made permutations to test the impact of the climatic factors as described in the Methods section.

**Table 2:** AIC selection at the transmission scale

Model	Number of parameters	AIC criterion
*a*(1), *b*(1), *c*(0), *d*(1), *α*(1)	5	1455.00
*a*(1), *b*(*R*), *c*(0), *d*(1), *α*(1)	15	1460.48
*a*(1), *b*(1), *c*(0), *d*(*R*), *α*(1)	15	1462.00
*a*(1), *b*(1), *c*(0), *d*(1), *α*(*Y*)	13	1468.29
*a*(1), *b*(1), *c*(0), *d*(1), *α*(*R*)	15	1471.00
*a*(1), *b*(1), *c*(0), *d*(*Y*), *α*(1)	13	1801.52
*a*(*R*), *b*(1), *c*(0), *d*(1), *α*(1)	15	1824.58

**Fig. 2:**
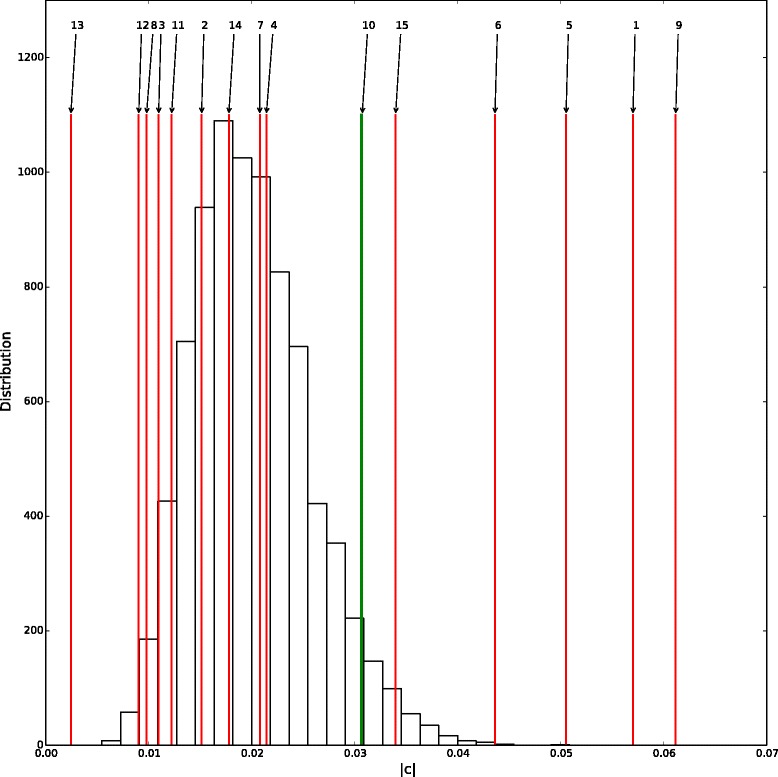
Theoretical distribution under the null hypothesis with the threshold (the 95th quantile) in green and the |c| values in red, standing for the climatic impacts of each factor estimated for the eleven regions and for the nine epidemic years (to 2003–2004 till 2012–2013 except 2009–2010) at the transmission scale. 1: Average temperature, 2: Daily variation of temperature, 3: Relative weekly variation of temperature, 4: Absolute weekly variation of temperature, 5: Average relative humidity, 6: Daily variation of relative humidity, 7: Relative weekly variation of relative humidity, 8: Absolute weekly variation of relative humidity, 9: Average absolute humidity, 10: Daily variation of absolute humidity, 11: Relative weekly variation of absolute humidity, 12: Absolute weekly variation of absolute humidity, 13: Average wind speed, 14: Rainfalls height, 15: Sunshine duration

**Fig. 3:**
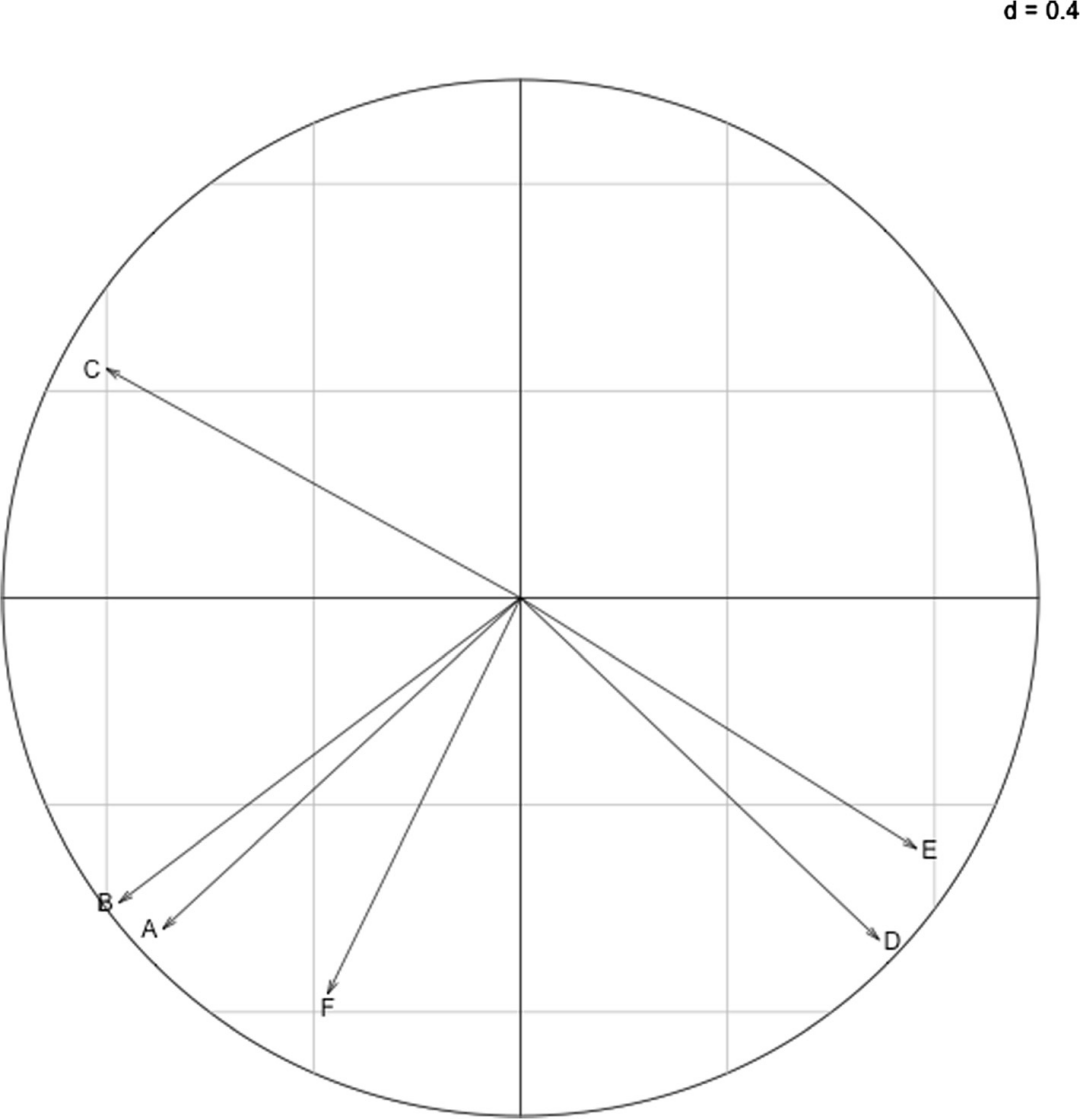
Correlation circle of the principal component analysis (PCA) on climatic data. A: Average temperature, B: Average absolute humidity, C: Average relative humidity, D: Daily variation of relative humidity, E: Sunshine duration, F: Daily variation of absolute humidity. The PCA explains 85.47 % of the variance with its first two axes explaining, respectively, 48.73 and 36.74 %

Six climatic factors appeared significant: the average absolute humidity, the average temperature, the average relative humidity, the daily variation of relative humidity, the sunshine duration and the daily variation of absolute humidity (see Fig. 2). The parameters and impacts of these climatic factors are summarized in Table 3. In order to search for confounding effects we built a principal component analysis (PCA) on the climatic data using R.3.1.2 [39] and the package ade4 [40–42]. The correlation circle of the PCA shows the correlations between variables (see Fig. 3). Two groups of variables are observed: on the one hand average temperature, average absolute humidity and diary variation of absolute humidity positively correlated and, on the other hand, average relative humidity negatively correlated with diary variation of relative humidity and sunshine duration.

**Table 3:** Global climatic impacts

Climatic factor	c	Standard deviation	Impact (%)
Average absolute humidity	−0.0612	0.0108	5.94
Average temperature	−0.0570	0.0109	5.54
Average relative humidity	−0.0505	0.0120	4.92
Daily variation of relative humidity	0.0436	0.0124	4.46
Sunshine duration	0.0340	0.0106	3.46
Daily variation of absolute humidity	−0.0307	0.0117	3.02

Besides the evaluation of impact of climatic factors at the transmission scale, the model built allowed the estimate of the susceptible population for an epidemic year $\widehat{N}$ with the definition of $\widehat{\alpha}$ that provides an estimate of the proportion of individuals who developed the disease in the pool of individuals that could have developed it. In the fifteen climatic models, estimates of *α* were included between 0.98 and 1 with a very low standard deviation (< 0.01).

### Impact of climatic factors at the epidemic scale

**Fig. 4:**
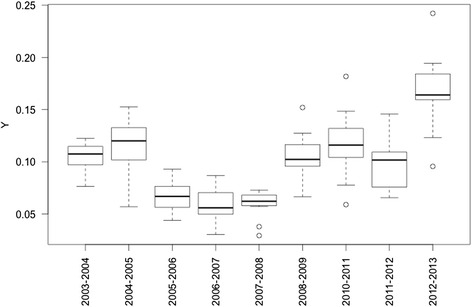
Boxplot of the ratio of cumulated number of infected individuals across the entire epidemic period to the total population (*Y*) of the eleven regions according to the nine epidemic years

Regional and seasonal variations appear in the epidemic size (see Fig. 4). In order to evaluate the impact of climatic factors on these variations we first chose a model according to the AIC criterion and second we built models with each climatic factor and tested the climatic impacts with permutations.

The auto-regressive coefficient *b* was not retained from the AIC selection procedure (see Table 4). That is why we chose a model only considering seasonal and regional variations to evaluate the impact of climatic factors.

**Table 4:** AIC selection at the epidemic scale

Model	Number of parameters	AIC criterion
*ES*_*Y*,*R*_ = *a*_0_ + *a*_*Y*_ + *a*_*R*_ + *b* ⋅ *ES*_*Y* − 1,*R*_	19	−241.64
*ES*_*Y*,*R*_ = *a*_0_ + *a*_*Y*_ + *a*_*R*_	18	−243.42
*ES*_*Y*,*R*_ = *a*_0_ + *a*_*Y*_	8	−225.85
*ES*_*Y*,*R*_ = *a*_0_ + *b* ⋅ *ES*_*Y* − 1,*R*_	2	−157.11
*ES*_*Y*,*R*_ = *a*_0_	1	−144.70
*ES*_*Y*,*R*_ = *a*_0_ + *a*_*Y*_ + *b* ⋅ *ES*_*Y* − 1,*R*_	9	−234.99
*ES*_*Y*,*R*_ = *a*_0_ + *a*_*R*_	11	−135.74

**Fig. 5:**
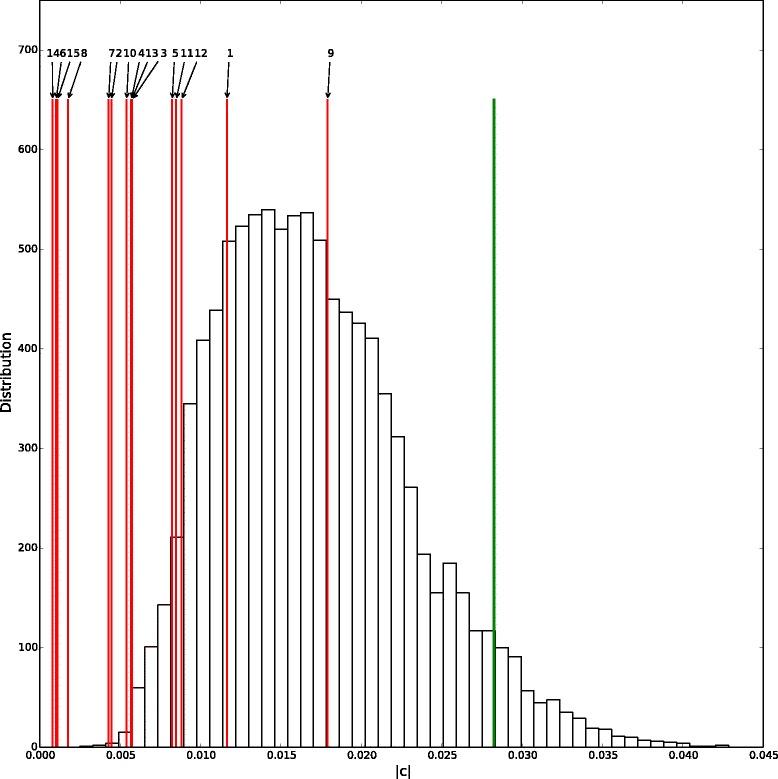
Theoretical distribution under the null hypothesis with the threshold (the 95th quantile) in green and the |c| values in red, standing for the climatic impacts of each factor estimated for the eleven regions and for the nine epidemic years (to 2003–2004 till 2012–2013 except 2009–2010) at the epidemic scale. 1: Average temperature, 2: Daily variation of temperature, 3: Relative weekly variation of temperature, 4: Absolute weekly variation of temperature, 5: Average relative humidity, 6: Daily variation of relative humidity, 7: Relative weekly variation of relative humidity, 8: Absolute weekly variation of relative humidity, 9: Average absolute humidity, 10: Daily variation of absolute humidity, 11: Relative weekly variation of absolute humidity, 12: Absolute weekly variation of absolute humidity, 13: Average wind speed, 14: Rainfalls height, 15: Sunshine duration

No climatic factors appeared significant at the epidemic scale (see Fig. 5) meaning that none of the climatic factors well explained the variation of epidemic size between regions and epidemic years.

Considering that variations in epidemic size could not be explained by our (measured) climatic variables, we then tried to decompose these variations into three sources. First variations in region characteristics (e.g., population size or non-measured climatic factors) can lead to systematic differences between regions. Second, temporal variations (e.g., in strain characteristics) can lead to systematic increase or decreased of epidemic sizes in all regions. Third, local conditions (in given epidemic years and regions) may also affect epidemic sizes. To quantify these three sources of variations, we built a model considering epidemic year and region as random variables: log(*ES*_*Y*,*R*_) = *a*_0_ + *a*_*Y*_ + *a*_*R*_ + *ε*, where *a*_*Y*_ (respectively *a*_*R*_) is distributed according to a Gaussian centered distribution with a standard deviation *σ*_*Y*_. (respectively *σ*_*R*_). *ε* stands for the residual variations, taking into account the local variations of a given epidemic year and region, it is distributed according to a Gaussian centered distribution with a standard deviation *σ*_*ε*_. The homoscedasticity of the residuals is shown in Additional file 2: Figure S1.

Parameters were estimated with R.3.1.2 [39] using the package lme4 [43, 44]. We found ${\widehat{\sigma}}_Y=0.036$, ${\widehat{\sigma}}_R=0.013$ d ${\widehat{\sigma}}_{\varepsilon }=0.0217$ meaning that variations from one epidemic year to another one, from one region to another one and due to local conditions account for 50.9, 18.4 and 30.7 %, respectively.

## Discussion

In the present paper, we presented the results of the analysis of the statistical link between influenza spread and fifteen climatic factors. Data were obtained from the French Réseau des GROG sentinel network. The network is based on voluntary practitioners who i) record acute respiratory infection and ii) randomly send nasal samples for an antigenic confirmation (or rejection) of influenza infection. Based on those two pieces of information, the Réseau des GROG sentinel network provides influenza incidence estimates of clinical cases. Two metrics were used for linking virus spread to climatic data: weekly incidence data of clinical cases and the epidemic size – measured as the total number of recorded clinical cases over the epidemic period.

Results of the analysis failed to isolate any correlation between epidemic size and climatic factors. Regarding weekly incidence data, we considered that incidence at time *t* was first affected by both the number of infected and susceptible individuals at time *t* − *1*, as it is classically assumed in epidemic dynamic models of infectious diseases [36]. Six climatic factors were found to be significantly linked to influenza spread: average temperature, average absolute and relative humidity, daily variations of absolute and relative humidity as well as sunshine duration. However, a principal component analysis revealed that upon these six factors, two groups of three highly correlated factors could be separated. On a practical point of view, this implies that within each of the two groups, it is likely that only one factor has a biological link to influenza spread, the two remaining factors being linked to the disease spread because they are linked to the first factor (confounding effect).

The first group of factors is made up of average temperature and absolute humidity, and daily variations of absolute humidity. The role of a cold and dry weather on influenza spread has been highlighted from laboratory studies [19, 20] and modeling approaches in temperate countries [13, 15, 16, 21] including France [45]. Moreover models that included weekly variations of both temperature and absolute humidity in Israel [46] and in New York City [47] predicted reliable influenza epidemic estimations (better estimations with both factors than only one). That is why both the average temperature and absolute humidity seem to play an important role on the influenza spread.

The second group of factors is made up of average relative humidity, daily variations of relative humidity and sunshine duration. Both laboratory [11] and simulation [14] studies enhanced the impact of the relative humidity. About sunshine duration, a decrease of sunshine might favor influenza spread [31] but surprisingly our results showed a positive impact of sunshine duration on influenza epidemic spread. That is why the average relative humidity might impact influenza spread whereas sunshine duration might be a confounding factor.

Overall, the impact of the significant factors remained relatively low (a few percent). This is not surprising when we compare our finding with what is found in the literature (3 % impact of absolute humidity in the Netherlands - [21], less than 2 % impact of both absolute humidity and temperature on influenza mortality in the USA - [15]). However, it is important to raise reasonable hypotheses for explaining why the impact of climatic factors is found so low. First, low impacts can arise from the presence of important noise in data. The Réseau des GROG sentinel network is based on a limited number of voluntary practitioners, leading to noise in incidence estimates. Second, in order to obtain relatively reliable incidence estimates, we had to average incidence over entire regions. Climate and disease spread can be disparate within a region, leading to weaken the link between climatic factors and disease spread. Third, the model, which has a lag of one week (linking incidence at time *t* with the number of susceptible and infected individuals at time *t* − *1*), can be a bit too simple. Actually simple compartmental models may not be sufficient to describe properly an influenza epidemic. Models are becoming more complex by, for example, taking into account more heterogeneous influenza transmission in the population (e.g., agent-based model) and including a contact network among people [48–50]. Finally, correlation between influenza spread and single climatic factors can be too simplistic. Climate can have a strong impact on disease spread, but on a more complex way involving several factors and potential interactions between these factors. Such combinations of factors were not considered in the model because it would have led to a huge number of hypotheses’ testing. Such an investigation of the most relevant combinations of climatic factors would be more relevantly achieved using descriptive statistics, but this was not the purpose of our study.

Another important question arising from our results is about of the disparity of the link of climatic factors with influenza spread using weekly incidence data and epidemic size data. The first obvious potential explanation is the lower statistical power associated to epidemic size data. Epidemic size is estimated only once per year while incidence is estimated every week. So epidemic size data contain less statistical information. An interesting alternative hypothesis could be that epidemic size and weekly incidence data capture different biological phenomena. Basically, incidence (corrected by the number of susceptible and infected individuals) may vary between weeks according to climatic factors for two reasons: i) because individuals are more susceptible to develop the clinical form of the infection and ii) because infection is more likely, i.e., the virus transmission rate increases. Epidemic size is schematically the result (product) of two phenomena: i) the proportion of individuals in the region that are susceptible to develop the clinical form of the disease upon infection and ii) the fraction of these individuals that will be reached by the virus, i.e., that will effectively become infected. If the latter phenomenon is linked to the virus transmission rate, the link is not linear. In particular, for large enough transmission rates, all susceptible individuals become infected during an epidemic and this term is poorly affected by the transmission rate. Interestingly, in that case, epidemic sizes are mainly an indicator of individuals’ susceptibility and hence contain information that differs from that of weekly incidence data.

The proportion of the susceptible (to the clinical disease) population that ultimately develops the disease is an important quantity for both data analysis interpretation and disease management. In data analysis, it will tell us how to interpret epidemic size data. When all susceptible individuals acquire the infection, then epidemic size is an indicator of the proportion of susceptible individuals in the population, i.e., the proportion of individuals that are in a healthy state (in terms of innate and acquire immunity) that does not permit them to control the disease upon infection. On a management point of view, if all individuals acquire the infection, this means that the virus transmission rate is high and reducing it will not necessarily lead to reduce its impact.

In our study, we introduced a term that we interpreted as the proportion of susceptible individuals who ultimately got infected. This is an interesting result, but which should be interpreted with great caution. First susceptibility is here defined as the ultimate development of the disease upon infection. It is hence not necessarily equivalent to susceptibility defined by antibody profiles. Second, it is important to recall that it is primarily a model parameter introduced for statistical convenience (i.e., a shape parameter). The fact that it equals one in our model only means that the decay in disease incidence at the end of the epidemic can be explained without having to assume any susceptible pool that would have escaped the infection. Since the study was not designed for estimating this biological quantity, we invite the reader not to interpret it as a formal estimation procedure of the proportion of susceptible individuals, but as a point raising interesting questions.

Several improvements could be brought to our analysis. First, it would be interesting to differentiate between the different subtypes of influenza. Influenza epidemics are often due to several subtypes that generate potentially shifted epidemics [51]. Practically, in our model this would imply that the number of susceptible individuals does not necessarily decreases with the cumulated number of influenza cases from all subtypes, but is subtype specific. Even though the use of permutation tests tends to reduce this problem, it would still be interesting to study the different subtypes separately because they might be differentially affected by climatic factors. Unfortunately, this information was not available in our data set.

The second interesting improvement that could be brought to our model is the consideration of different age-classes. Indeed, influenza is known to spread differentially within and between age-classes [52–54]. However, introducing age-classes in our model would tend to make it more complex. In the current paper we adopted a practical point of view by considering only the global spread of the epidemic without considering the heterogeneity of individuals that may exist within a population (age-classes, social classes, job-dependent degree of exposure, etc.).

## Conclusion

Proper modeling of the relationship between climatic variables and infectious diseases spread and impact presents a challenging task. We presented a way to conciliate statistical and dynamical models of infectious diseases in a way that keeps the simplicity of statistical approach while introducing key knowledge about infectious dynamics (such as the decay of incidence after the epidemic peak).

We performed our study on two important influenza response variables at two levels: intra- and inter-annually. Linking variations of weekly incidence data with climatic factors is relevant because it allows anticipating the decay or increase in the number of cases of influenza in the weeks to come. The epidemic size is also a very important measure because it allows quantifying the impact of influenza according to climatic factors. This is especially valuable in the context of global climate changes to anticipate the future impact of influenza.

## Additional files

Additional file 1: Meteorological data description. (PDF 211 kb) Additional file 2: Figure of the homoscedasticity of the residuals of the model at the epidemic scale. (PDF 97 kb) Additional file 3: Epidemiological data. (PDF 136 kb)
