# Supplementary material for: Quantifying the role of weather on seasonal influenza
Source: BMC Public Health. 2016 May 26;16:441. doi: 10.1186/s12889-016-3114-x (PMC4881007; doi:10.1186/s12889-016-3114-x)
Supplement: Additional file 1: — Meteorological data description. (PDF 211 kb) [file 12889_2016_3114_MOESM1_ESM.pdf]

Additional Material 1: Meteorological data

Meteorological parameter	Unity	Definition
Rainfalls height	mm	Rainfalls height collected between 6 am the D-Day and 6 am the following day.
Minimal temperature	C°	Minimal temperature collected under shelter between 6 am the day before and 6 am the D-Day.
Maximal temperature	C°	Maximal temperature collected under shelter between 6 am the day before and 6 am the D-Day.
Average temperature	C°	Average of 24 hourly values (from 1 am to 12 pm) of air temperature collected under shelter.
Wind speed	m/s	Average of 24 hourly values (from 1 am to 12 pm) of wind speed averaged over 10 minutes (measure height: 10 m).
Minimal relative humidity	%	Minimum of relative humidity collected under shelter between 0 am the D-Day and 0 am the day after.
Maximal relative humidity	%	Maximum of relative humidity collected under shelter between 0 am the D-Day and 0 am the day after.
Average relative Humidity[1]	%	Average of 24 hourly values (from 1 am to 12 pm) of relative humidity collected under shelter.
Minimal absolute humidity	g/m^3	Minimum of absolute humidity collected under shelter between 0 am the D-Day and 0 am the day after.
Maximal Absolute Humidity	g/m^3	Maximum of absolute humidity collected under shelter between 0 am the D-Day and 0 am the day after.
Average absolute humidity[2]	g/m^3	Average of 24 hourly values (from 1 am to 12 pm) of absolute humidity collected under shelter.
Sunshine duration	minutes	Duration between 0 am and 12 pm during which solar radiation is of sufficient intensity ($> 120W/m^{-2}$) to produce distinct shadows.

[1] Relative humidity is the ratio of the actual amount of water vapor contained in the air to the maximum amount that the air can hold according to its temperature.
[2] Absolute humidity is the amount of water vapor contained in the air.
