# Supplementary material for: Quantifying the role of weather on seasonal influenza
Source: BMC Public Health. 2016 May 26;16:441. doi: 10.1186/s12889-016-3114-x (PMC4881007; doi:10.1186/s12889-016-3114-x)
Supplement: Additional file 2: — Figure of the homoscedasticity of the residuals of the model at the epidemic scale. (PDF 97 kb) [file 12889_2016_3114_MOESM2_ESM.pdf]

Additional Material 2: The homoscedasticity of the residuals

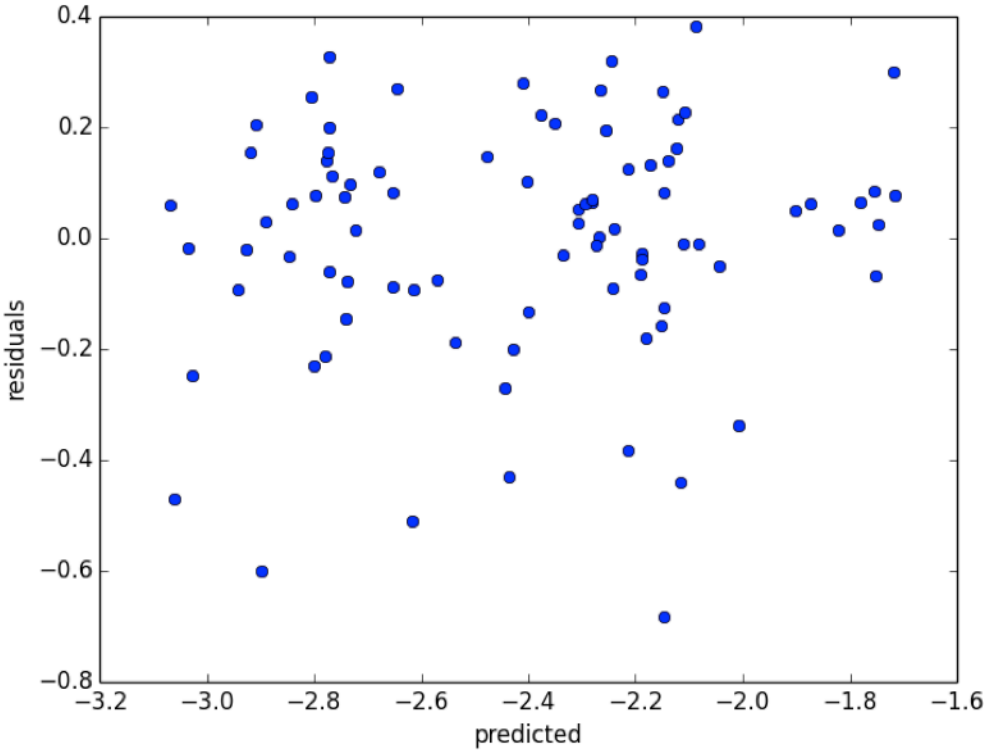

Figure S1 - Residuals versus predicted values of the model built at the epidemic scale explaining the logarithm of the epidemic size according to epidemic year and region as random variables.
