# Supplementary material for: Quantifying the role of weather on seasonal influenza
Source: BMC Public Health. 2016 May 26;16:441. doi: 10.1186/s12889-016-3114-x (PMC4881007; doi:10.1186/s12889-016-3114-x)
Supplement: Additional file 3: — Epidemiological data. (PDF 136 kb) [file 12889_2016_3114_MOESM3_ESM.pdf]

Region	ID_Week (year+number)	Incidence (per 100000 inhabitants)
Aquitaine	200341	NA
Aquitaine	200342	0
Aquitaine	200343	109.2727
Aquitaine	200344	225.1212
Aquitaine	200345	517.3975
Aquitaine	200346	632.6666
Aquitaine	200347	1234.293
Aquitaine	200348	1735.625
Aquitaine	200349	2218.8
Aquitaine	200350	1975.8
Aquitaine	200351	1095.611
Aquitaine	200352	504
Aquitaine	200401	87.41667
Aquitaine	200402	0
Aquitaine	200403	0
Aquitaine	200404	0
Aquitaine	200405	NA
Aquitaine	200406	NA
Aquitaine	200407	NA
Aquitaine	200408	NA
Aquitaine	200409	NA
Aquitaine	200410	NA
Aquitaine	200411	NA
Aquitaine	200412	NA
Aquitaine	200413	NA
Aquitaine	200414	NA
Aquitaine	200441	0
Aquitaine	200442	0
Aquitaine	200443	0
Aquitaine	200444	0
Aquitaine	200445	0
Aquitaine	200446	0
Aquitaine	200447	0
Aquitaine	200448	0
Aquitaine	200449	0
Aquitaine	200450	0
Aquitaine	200451	0
Aquitaine	200452	201.1111
Aquitaine	200453	240.4444
Aquitaine	200501	425.8111
Aquitaine	200502	577.7273
Aquitaine	200503	1100.879
Aquitaine	200504	1531.561
Aquitaine	200505	2067.6
Aquitaine	200506	2271.471

Aquitaine	200507	2308.602
Aquitaine	200508	1448.495
Aquitaine	200509	876.8619
Aquitaine	200510	704.0444
Aquitaine	200511	704.6667
Aquitaine	200512	NA
Aquitaine	200513	NA
Aquitaine	200514	NA
Aquitaine	200541	NA
Aquitaine	200542	NA
Aquitaine	200543	0
Aquitaine	200544	0
Aquitaine	200545	0
Aquitaine	200546	0
Aquitaine	200547	0
Aquitaine	200548	0
Aquitaine	200549	0
Aquitaine	200550	0
Aquitaine	200551	186.6667
Aquitaine	200552	191.1111
Aquitaine	200601	174
Aquitaine	200602	373.75
Aquitaine	200603	639.1666
Aquitaine	200604	1097.087
Aquitaine	200605	999.6429
Aquitaine	200606	841.4603
Aquitaine	200607	762.4
Aquitaine	200608	449.0667
Aquitaine	200609	476.4
Aquitaine	200610	875.5546
Aquitaine	200611	1016.167
Aquitaine	200612	1217.466
Aquitaine	200613	1012.032
Aquitaine	200614	557.3333
Aquitaine	200641	NA
Aquitaine	200642	0
Aquitaine	200643	0
Aquitaine	200644	0
Aquitaine	200645	0
Aquitaine	200646	0
Aquitaine	200647	104.4667
Aquitaine	200648	130.8667
Aquitaine	200649	118.8
Aquitaine	200650	0
Aquitaine	200651	0
Aquitaine	200652	0

Aquitaine	200701	397.4
Aquitaine	200702	462
Aquitaine	200703	881.7926
Aquitaine	200704	978.824
Aquitaine	200705	1773.981
Aquitaine	200706	2572.4
Aquitaine	200707	1563.794
Aquitaine	200708	917.1556
Aquitaine	200709	NA
Aquitaine	200710	NA
Aquitaine	200711	NA
Aquitaine	200712	NA
Aquitaine	200713	NA
Aquitaine	200714	NA
Aquitaine	200741	0
Aquitaine	200742	0
Aquitaine	200743	0
Aquitaine	200744	0
Aquitaine	200745	0
Aquitaine	200746	0
Aquitaine	200747	0
Aquitaine	200748	152.5833
Aquitaine	200749	309.3
Aquitaine	200750	325.35
Aquitaine	200751	460.6222
Aquitaine	200752	835.7778
Aquitaine	200801	465.7778
Aquitaine	200802	534.4445
Aquitaine	200803	443.7859
Aquitaine	200804	879.0889
Aquitaine	200805	1219.728
Aquitaine	200806	1313.5
Aquitaine	200807	1514.917
Aquitaine	200808	760
Aquitaine	200809	451.7333
Aquitaine	200810	279.5333
Aquitaine	200811	484.8
Aquitaine	200812	NA
Aquitaine	200813	NA
Aquitaine	200814	NA
Aquitaine	200841	0
Aquitaine	200842	0
Aquitaine	200843	0
Aquitaine	200844	0
Aquitaine	200845	0
Aquitaine	200846	0

Aquitaine	200847	0
Aquitaine	200848	0
Aquitaine	200849	180.4444
Aquitaine	200850	412.4
Aquitaine	200851	991.8
Aquitaine	200852	1063.822
Aquitaine	200901	196.7399
Aquitaine	200902	1576.677
Aquitaine	200903	2040.764
Aquitaine	200904	1951.295
Aquitaine	200905	1392.857
Aquitaine	200906	1087.422
Aquitaine	200907	521.0528
Aquitaine	200908	764.75
Aquitaine	200909	388.125
Aquitaine	200910	535.5
Aquitaine	200911	452.6667
Aquitaine	200912	710.0001
Aquitaine	200913	674.3334
Aquitaine	200914	NA
Aquitaine	201041	0
Aquitaine	201042	NA
Aquitaine	201043	NA
Aquitaine	201044	NA
Aquitaine	201045	NA
Aquitaine	201046	NA
Aquitaine	201047	0
Aquitaine	201048	0
Aquitaine	201049	120.3333
Aquitaine	201050	290.85
Aquitaine	201051	264.6
Aquitaine	201052	231.3667
Aquitaine	201101	772.6889
Aquitaine	201102	894.2
Aquitaine	201103	1258.948
Aquitaine	201104	811.2674
Aquitaine	201105	669.8269
Aquitaine	201106	127.125
Aquitaine	201107	456.25
Aquitaine	201108	NA
Aquitaine	201109	NA
Aquitaine	201110	NA
Aquitaine	201111	112.2222
Aquitaine	201112	108
Aquitaine	201113	132.3333
Aquitaine	201114	NA

Aquitaine	201141	NA
Aquitaine	201142	0
Aquitaine	201143	0
Aquitaine	201144	0
Aquitaine	201145	0
Aquitaine	201146	89.57143
Aquitaine	201147	92.09524
Aquitaine	201148	89.47619
Aquitaine	201149	0
Aquitaine	201150	NA
Aquitaine	201151	NA
Aquitaine	201152	NA
Aquitaine	201201	0
Aquitaine	201202	162.5
Aquitaine	201203	202.6667
Aquitaine	201204	261.5667
Aquitaine	201205	292
Aquitaine	201206	1046.229
Aquitaine	201207	1938.886
Aquitaine	201208	1955.357
Aquitaine	201209	1623.611
Aquitaine	201210	1190.139
Aquitaine	201211	877
Aquitaine	201212	650.3111
Aquitaine	201213	439.4667
Aquitaine	201214	199.85
Aquitaine	201241	NA
Aquitaine	201242	NA
Aquitaine	201243	0
Aquitaine	201244	0
Aquitaine	201245	0
Aquitaine	201246	252
Aquitaine	201247	264.2222
Aquitaine	201248	578.7302
Aquitaine	201249	547.5619
Aquitaine	201250	1165.5
Aquitaine	201251	1551.044
Aquitaine	201252	1304.5
Aquitaine	201301	802.2
Aquitaine	201302	921.8223
Aquitaine	201303	738.2667
Aquitaine	201304	1400.8
Aquitaine	201305	2196.6
Aquitaine	201306	1400.5
Aquitaine	201307	2383.383
Aquitaine	201308	2826.75

Aquitaine	201309	1320.7
Aquitaine	201310	NA
Aquitaine	201311	NA
Aquitaine	201312	NA
Aquitaine	201313	NA
Aquitaine	201314	NA
Lower Normandy	200341	0
Lower Normandy	200342	0
Lower Normandy	200343	75.80952
Lower Normandy	200344	63.71429
Lower Normandy	200345	216.1889
Lower Normandy	200346	501.7496
Lower Normandy	200347	1367.147
Lower Normandy	200348	1755.762
Lower Normandy	200349	2247.477
Lower Normandy	200350	1994.238
Lower Normandy	200351	1838.419
Lower Normandy	200352	666.269
Lower Normandy	200401	365.3333
Lower Normandy	200402	109.4667
Lower Normandy	200403	89.13334
Lower Normandy	200404	0
Lower Normandy	200405	0
Lower Normandy	200406	0
Lower Normandy	200407	0
Lower Normandy	200408	0
Lower Normandy	200409	0
Lower Normandy	200410	0
Lower Normandy	200411	0
Lower Normandy	200412	0
Lower Normandy	200413	NA
Lower Normandy	200414	NA
Lower Normandy	200441	0
Lower Normandy	200442	0
Lower Normandy	200443	0
Lower Normandy	200444	0
Lower Normandy	200445	0
Lower Normandy	200446	0
Lower Normandy	200447	0
Lower Normandy	200448	0
Lower Normandy	200449	0
Lower Normandy	200450	0
Lower Normandy	200451	0
Lower Normandy	200452	120.8889
Lower Normandy	200453	173.963
Lower Normandy	200501	266.7104

Lower Normandy	200502	278.7738
Lower Normandy	200503	429.5272
Lower Normandy	200504	583.9719
Lower Normandy	200505	826.3167
Lower Normandy	200506	1370.083
Lower Normandy	200507	1408.036
Lower Normandy	200508	909.8773
Lower Normandy	200509	711.5594
Lower Normandy	200510	1064.463
Lower Normandy	200511	995.6
Lower Normandy	200512	953.7667
Lower Normandy	200513	788.3333
Lower Normandy	200514	483.6333
Lower Normandy	200541	0
Lower Normandy	200542	0
Lower Normandy	200543	0
Lower Normandy	200544	0
Lower Normandy	200545	26.16667
Lower Normandy	200546	30.19444
Lower Normandy	200547	41.75
Lower Normandy	200548	0
Lower Normandy	200549	0
Lower Normandy	200550	27.65
Lower Normandy	200551	26.51667
Lower Normandy	200552	27.91667
Lower Normandy	200601	37.41026
Lower Normandy	200602	90.76923
Lower Normandy	200603	201.1218
Lower Normandy	200604	361.2647
Lower Normandy	200605	900.4064
Lower Normandy	200606	1208.629
Lower Normandy	200607	1691.3
Lower Normandy	200608	952.1143
Lower Normandy	200609	480.675
Lower Normandy	200610	282.9917
Lower Normandy	200611	436.875
Lower Normandy	200612	376.4
Lower Normandy	200613	265.3
Lower Normandy	200614	137.55
Lower Normandy	200641	NA
Lower Normandy	200642	NA
Lower Normandy	200643	0
Lower Normandy	200644	0
Lower Normandy	200645	0
Lower Normandy	200646	0
Lower Normandy	200647	0

Lower Normandy	200648	0
Lower Normandy	200649	0
Lower Normandy	200650	0
Lower Normandy	200651	79.91667
Lower Normandy	200652	297.675
Lower Normandy	200701	310.4222
Lower Normandy	200702	397.4857
Lower Normandy	200703	395.9471
Lower Normandy	200704	516.4161
Lower Normandy	200705	867.6373
Lower Normandy	200706	1330.836
Lower Normandy	200707	1316.875
Lower Normandy	200708	700.825
Lower Normandy	200709	268.4083
Lower Normandy	200710	319
Lower Normandy	200711	136.4286
Lower Normandy	200712	303.6429
Lower Normandy	200713	NA
Lower Normandy	200714	NA
Lower Normandy	200741	0
Lower Normandy	200742	0
Lower Normandy	200743	0
Lower Normandy	200744	39.54167
Lower Normandy	200745	43.79167
Lower Normandy	200746	38.95834
Lower Normandy	200747	0
Lower Normandy	200748	0
Lower Normandy	200749	68.47619
Lower Normandy	200750	241.2699
Lower Normandy	200751	304.14
Lower Normandy	200752	339.3464
Lower Normandy	200801	314.1448
Lower Normandy	200802	371.1615
Lower Normandy	200803	441.1397
Lower Normandy	200804	552.4522
Lower Normandy	200805	522.8334
Lower Normandy	200806	562.2783
Lower Normandy	200807	669.4802
Lower Normandy	200808	645.3402
Lower Normandy	200809	389.2034
Lower Normandy	200810	368.5662
Lower Normandy	200811	350.4306
Lower Normandy	200812	444
Lower Normandy	200813	325.8333
Lower Normandy	200814	313.3333
Lower Normandy	200841	NA

Lower Normandy	200842	0
Lower Normandy	200843	0
Lower Normandy	200844	0
Lower Normandy	200845	0
Lower Normandy	200846	0
Lower Normandy	200847	0
Lower Normandy	200848	0
Lower Normandy	200849	20.68254
Lower Normandy	200850	155.1631
Lower Normandy	200851	416.2014
Lower Normandy	200852	691.6075
Lower Normandy	200901	887.1705
Lower Normandy	200902	1028.968
Lower Normandy	200903	1225.199
Lower Normandy	200904	1196.51
Lower Normandy	200905	613.9216
Lower Normandy	200906	348.2157
Lower Normandy	200907	77.16666
Lower Normandy	200908	0
Lower Normandy	200909	0
Lower Normandy	200910	0
Lower Normandy	200911	0
Lower Normandy	200912	NA
Lower Normandy	200913	NA
Lower Normandy	200914	NA
Lower Normandy	201041	484.4
Lower Normandy	201042	257.3333
Lower Normandy	201043	0
Lower Normandy	201044	0
Lower Normandy	201045	27.44444
Lower Normandy	201046	113.9704
Lower Normandy	201047	158.0176
Lower Normandy	201048	327.8036
Lower Normandy	201049	561.756
Lower Normandy	201050	908.0372
Lower Normandy	201051	1314.561
Lower Normandy	201052	1420.662
Lower Normandy	201101	1661.412
Lower Normandy	201102	1468.526
Lower Normandy	201103	1287.718
Lower Normandy	201104	951.707
Lower Normandy	201105	936.4561
Lower Normandy	201106	589.1534
Lower Normandy	201107	366.8926
Lower Normandy	201108	132.4296
Lower Normandy	201109	91.33334

Lower Normandy	201110	0
Lower Normandy	201111	0
Lower Normandy	201112	NA
Lower Normandy	201113	NA
Lower Normandy	201114	NA
Lower Normandy	201141	NA
Lower Normandy	201142	0
Lower Normandy	201143	NA
Lower Normandy	201144	NA
Lower Normandy	201145	NA
Lower Normandy	201146	NA
Lower Normandy	201147	0
Lower Normandy	201148	0
Lower Normandy	201149	0
Lower Normandy	201150	0
Lower Normandy	201151	0
Lower Normandy	201152	0
Lower Normandy	201201	56.06667
Lower Normandy	201202	60.92778
Lower Normandy	201203	147.0722
Lower Normandy	201204	173.0333
Lower Normandy	201205	378.8485
Lower Normandy	201206	564.5727
Lower Normandy	201207	844.0455
Lower Normandy	201208	966.5508
Lower Normandy	201209	807.7674
Lower Normandy	201210	890.6275
Lower Normandy	201211	681.5
Lower Normandy	201212	579.5
Lower Normandy	201213	529.9259
Lower Normandy	201214	NA
Lower Normandy	201241	0
Lower Normandy	201242	0
Lower Normandy	201243	0
Lower Normandy	201244	0
Lower Normandy	201245	102.9167
Lower Normandy	201246	94.33334
Lower Normandy	201247	108.25
Lower Normandy	201248	0
Lower Normandy	201249	171.8095
Lower Normandy	201250	339.4445
Lower Normandy	201251	429.4603
Lower Normandy	201252	480.127
Lower Normandy	201301	368.0238
Lower Normandy	201302	475.5952
Lower Normandy	201303	439.3158

Lower Normandy	201304	749.1843
Lower Normandy	201305	1065.184
Lower Normandy	201306	1239.912
Lower Normandy	201307	1002.914
Lower Normandy	201308	1012.088
Lower Normandy	201309	1104.992
Lower Normandy	201310	852.7619
Lower Normandy	201311	292.8095
Lower Normandy	201312	127.8333
Lower Normandy	201313	0
Lower Normandy	201314	99.73334
Brittany	200341	34.48718
Brittany	200342	208.3077
Brittany	200343	254.31
Brittany	200344	324.4424
Brittany	200345	694.8563
Brittany	200346	1176.167
Brittany	200347	1481.626
Brittany	200348	1913.974
Brittany	200349	2125.401
Brittany	200350	1658.429
Brittany	200351	1517.5
Brittany	200352	535.2778
Brittany	200401	344.6667
Brittany	200402	0
Brittany	200403	0
Brittany	200404	0
Brittany	200405	0
Brittany	200406	NA
Brittany	200407	NA
Brittany	200408	NA
Brittany	200409	NA
Brittany	200410	NA
Brittany	200411	NA
Brittany	200412	NA
Brittany	200413	NA
Brittany	200414	NA
Brittany	200441	0
Brittany	200442	0
Brittany	200443	0
Brittany	200444	0
Brittany	200445	0
Brittany	200446	0
Brittany	200447	0
Brittany	200448	0
Brittany	200449	0

Brittany	200450	0
Brittany	200451	90.25
Brittany	200452	189.7778
Brittany	200453	437.1111
Brittany	200501	303.5397
Brittany	200502	381.0714
Brittany	200503	1049.396
Brittany	200504	1403.934
Brittany	200505	1763.148
Brittany	200506	NA
Brittany	200507	NA
Brittany	200508	NA
Brittany	200509	525.8667
Brittany	200510	683.2778
Brittany	200511	665.3334
Brittany	200512	905
Brittany	200513	458
Brittany	200514	229.3333
Brittany	200541	0
Brittany	200542	0
Brittany	200543	0
Brittany	200544	0
Brittany	200545	0
Brittany	200546	0
Brittany	200547	0
Brittany	200548	0
Brittany	200549	0
Brittany	200550	127.037
Brittany	200551	132.8704
Brittany	200552	426.4167
Brittany	200601	331.6786
Brittany	200602	347.5476
Brittany	200603	447.8571
Brittany	200604	589.314
Brittany	200605	806.9063
Brittany	200606	977.246
Brittany	200607	1020.7
Brittany	200608	1108.326
Brittany	200609	689.8466
Brittany	200610	649.7582
Brittany	200611	476.3518
Brittany	200612	365.9722
Brittany	200613	190.7955
Brittany	200614	191.1364
Brittany	200641	0
Brittany	200642	0

Brittany	200643	0
Brittany	200644	0
Brittany	200645	0
Brittany	200646	97.08334
Brittany	200647	128.8333
Brittany	200648	189.6667
Brittany	200649	153.1429
Brittany	200650	236.8452
Brittany	200651	409.2024
Brittany	200652	338.4954
Brittany	200701	359.3664
Brittany	200702	419.0972
Brittany	200703	689.1786
Brittany	200704	753.5715
Brittany	200705	1205.88
Brittany	200706	1818.788
Brittany	200707	1373.263
Brittany	200708	470.8658
Brittany	200709	604.3889
Brittany	200710	631
Brittany	200711	581
Brittany	200712	325.5556
Brittany	200713	NA
Brittany	200714	NA
Brittany	200741	0
Brittany	200742	0
Brittany	200743	0
Brittany	200744	0
Brittany	200745	0
Brittany	200746	0
Brittany	200747	0
Brittany	200748	0
Brittany	200749	74.14815
Brittany	200750	199.0074
Brittany	200751	289.9344
Brittany	200752	339.4286
Brittany	200801	334.9881
Brittany	200802	507.5805
Brittany	200803	432.0658
Brittany	200804	732.126
Brittany	200805	1024.413
Brittany	200806	1118.571
Brittany	200807	1091.677
Brittany	200808	1077.288
Brittany	200809	744.5387
Brittany	200810	483.4259

Brittany	200811	638.7333
Brittany	200812	762
Brittany	200813	1224.578
Brittany	200814	882
Brittany	200841	0
Brittany	200842	0
Brittany	200843	82.60001
Brittany	200844	55.06667
Brittany	200845	218.6667
Brittany	200846	78.33334
Brittany	200847	92.06667
Brittany	200848	0
Brittany	200849	149.0833
Brittany	200850	501.5
Brittany	200851	862.2
Brittany	200852	1166.409
Brittany	200901	2386.094
Brittany	200902	1919.284
Brittany	200903	1889.482
Brittany	200904	1889.755
Brittany	200905	1147.771
Brittany	200906	459.2699
Brittany	200907	182
Brittany	200908	120.3333
Brittany	200909	220.7333
Brittany	200910	271.1333
Brittany	200911	445.6
Brittany	200912	698.25
Brittany	200913	599.8572
Brittany	200914	442.8571
Brittany	201041	NA
Brittany	201042	0
Brittany	201043	0
Brittany	201044	367
Brittany	201045	374.5833
Brittany	201046	825.9722
Brittany	201047	304.8889
Brittany	201048	157.6667
Brittany	201049	160.0833
Brittany	201050	410.25
Brittany	201051	513
Brittany	201052	793.5577
Brittany	201101	944.5641
Brittany	201102	912.9231
Brittany	201103	988.022
Brittany	201104	1482.781

Brittany	201105	1584.525
Brittany	201106	1027.841
Brittany	201107	1101.513
Brittany	201108	570
Brittany	201109	318.5
Brittany	201110	327.0833
Brittany	201111	NA
Brittany	201112	NA
Brittany	201113	NA
Brittany	201114	NA
Brittany	201141	NA
Brittany	201142	0
Brittany	201143	0
Brittany	201144	NA
Brittany	201145	NA
Brittany	201146	NA
Brittany	201147	NA
Brittany	201148	NA
Brittany	201149	NA
Brittany	201150	457.9259
Brittany	201151	453.4444
Brittany	201152	554.3334
Brittany	201201	382.6667
Brittany	201202	626.8
Brittany	201203	650.1
Brittany	201204	646
Brittany	201205	654.5
Brittany	201206	886.6
Brittany	201207	822.3857
Brittany	201208	933.2143
Brittany	201209	1130.556
Brittany	201210	1605.333
Brittany	201211	1566.667
Brittany	201212	1541.333
Brittany	201213	1296.078
Brittany	201214	974.7778
Brittany	201241	NA
Brittany	201242	NA
Brittany	201243	NA
Brittany	201244	0
Brittany	201245	0
Brittany	201246	0
Brittany	201247	0
Brittany	201248	88.53334
Brittany	201249	532.4222
Brittany	201250	636

Brittany	201251	1101.222
Brittany	201252	823.0222
Brittany	201301	1497.645
Brittany	201302	640.1
Brittany	201303	832.7083
Brittany	201304	932.9375
Brittany	201305	1648.813
Brittany	201306	2328.125
Brittany	201307	2007.365
Brittany	201308	1763.636
Brittany	201309	1333.682
Brittany	201310	862.7936
Brittany	201311	646.5912
Brittany	201312	377.3611
Brittany	201313	768.7778
Brittany	201314	908.6667
Upper Normandy	200341	NA
Upper Normandy	200342	0
Upper Normandy	200343	351.1667
Upper Normandy	200344	360.1667
Upper Normandy	200345	1410
Upper Normandy	200346	1375.429
Upper Normandy	200347	2209.086
Upper Normandy	200348	2381.968
Upper Normandy	200349	1044.394
Upper Normandy	200350	1429.988
Upper Normandy	200351	890.7381
Upper Normandy	200352	NA
Upper Normandy	200401	NA
Upper Normandy	200402	NA
Upper Normandy	200403	NA
Upper Normandy	200404	NA
Upper Normandy	200405	NA
Upper Normandy	200406	NA
Upper Normandy	200407	NA
Upper Normandy	200408	NA
Upper Normandy	200409	NA
Upper Normandy	200410	NA
Upper Normandy	200411	NA
Upper Normandy	200412	NA
Upper Normandy	200413	NA
Upper Normandy	200414	NA
Upper Normandy	200441	NA
Upper Normandy	200442	NA
Upper Normandy	200443	NA
Upper Normandy	200444	NA

Upper Normandy	200445	NA
Upper Normandy	200446	NA
Upper Normandy	200447	NA
Upper Normandy	200448	144.1667
Upper Normandy	200449	141.25
Upper Normandy	200450	141.0833
Upper Normandy	200451	0
Upper Normandy	200452	0
Upper Normandy	200453	235.2778
Upper Normandy	200501	410.6667
Upper Normandy	200502	NA
Upper Normandy	200503	NA
Upper Normandy	200504	NA
Upper Normandy	200505	1328.492
Upper Normandy	200506	1355.032
Upper Normandy	200507	3057.786
Upper Normandy	200508	1293.238
Upper Normandy	200509	1650.276
Upper Normandy	200510	1633.776
Upper Normandy	200511	NA
Upper Normandy	200512	NA
Upper Normandy	200513	NA
Upper Normandy	200514	NA
Upper Normandy	200541	0
Upper Normandy	200542	0
Upper Normandy	200543	NA
Upper Normandy	200544	NA
Upper Normandy	200545	NA
Upper Normandy	200546	NA
Upper Normandy	200547	0
Upper Normandy	200548	0
Upper Normandy	200549	0
Upper Normandy	200550	0
Upper Normandy	200551	0
Upper Normandy	200552	0
Upper Normandy	200601	388.3333
Upper Normandy	200602	NA
Upper Normandy	200603	NA
Upper Normandy	200604	NA
Upper Normandy	200605	1041.911
Upper Normandy	200606	NA
Upper Normandy	200607	NA
Upper Normandy	200608	NA
Upper Normandy	200609	1444
Upper Normandy	200610	1865.6
Upper Normandy	200611	1423.5

Upper Normandy	200612	1884.875
Upper Normandy	200613	1440
Upper Normandy	200614	1604.667
Upper Normandy	200641	NA
Upper Normandy	200642	0
Upper Normandy	200643	0
Upper Normandy	200644	0
Upper Normandy	200645	0
Upper Normandy	200646	0
Upper Normandy	200647	0
Upper Normandy	200648	0
Upper Normandy	200649	0
Upper Normandy	200650	0
Upper Normandy	200651	NA
Upper Normandy	200652	NA
Upper Normandy	200701	NA
Upper Normandy	200702	291.3333
Upper Normandy	200703	263.581
Upper Normandy	200704	955.4857
Upper Normandy	200705	773.6349
Upper Normandy	200706	1421.69
Upper Normandy	200707	2465.841
Upper Normandy	200708	1543.524
Upper Normandy	200709	323.1667
Upper Normandy	200710	1094.333
Upper Normandy	200711	NA
Upper Normandy	200712	NA
Upper Normandy	200713	NA
Upper Normandy	200714	NA
Upper Normandy	200741	NA
Upper Normandy	200742	0
Upper Normandy	200743	0
Upper Normandy	200744	0
Upper Normandy	200745	0
Upper Normandy	200746	0
Upper Normandy	200747	122.5556
Upper Normandy	200748	277.0833
Upper Normandy	200749	414.3611
Upper Normandy	200750	303.3333
Upper Normandy	200751	NA
Upper Normandy	200752	NA
Upper Normandy	200801	NA
Upper Normandy	200802	532.1334
Upper Normandy	200803	660.9166
Upper Normandy	200804	833.4
Upper Normandy	200805	1063.431

Upper Normandy	200806	896.3428
Upper Normandy	200807	1346.667
Upper Normandy	200808	539.6667
Upper Normandy	200809	545
Upper Normandy	200810	85.5
Upper Normandy	200811	93.41667
Upper Normandy	200812	441.3333
Upper Normandy	200813	767.5
Upper Normandy	200814	1402.5
Upper Normandy	200841	NA
Upper Normandy	200842	NA
Upper Normandy	200843	NA
Upper Normandy	200844	NA
Upper Normandy	200845	0
Upper Normandy	200846	0
Upper Normandy	200847	0
Upper Normandy	200848	0
Upper Normandy	200849	307.4445
Upper Normandy	200850	713.8889
Upper Normandy	200851	1643.889
Upper Normandy	200852	1327.944
Upper Normandy	200901	1881
Upper Normandy	200902	1354.667
Upper Normandy	200903	1093.333
Upper Normandy	200904	1160.956
Upper Normandy	200905	493.5333
Upper Normandy	200906	577.8
Upper Normandy	200907	241.8333
Upper Normandy	200908	325.25
Upper Normandy	200909	91.66667
Upper Normandy	200910	125.8333
Upper Normandy	200911	0
Upper Normandy	200912	NA
Upper Normandy	200913	NA
Upper Normandy	200914	NA
Upper Normandy	201041	NA
Upper Normandy	201042	NA
Upper Normandy	201043	NA
Upper Normandy	201044	NA
Upper Normandy	201045	NA
Upper Normandy	201046	NA
Upper Normandy	201047	187.875
Upper Normandy	201048	726.375
Upper Normandy	201049	1281.369
Upper Normandy	201050	2112.232
Upper Normandy	201051	1688.036

Upper Normandy	201052	1978.38
Upper Normandy	201101	1288.889
Upper Normandy	201102	957
Upper Normandy	201103	985.0769
Upper Normandy	201104	1436.688
Upper Normandy	201105	1120.144
Upper Normandy	201106	1204.8
Upper Normandy	201107	799.5
Upper Normandy	201108	NA
Upper Normandy	201109	NA
Upper Normandy	201110	NA
Upper Normandy	201111	NA
Upper Normandy	201112	NA
Upper Normandy	201113	NA
Upper Normandy	201114	NA
Upper Normandy	201141	NA
Upper Normandy	201142	NA
Upper Normandy	201143	NA
Upper Normandy	201144	NA
Upper Normandy	201145	0
Upper Normandy	201146	NA
Upper Normandy	201147	NA
Upper Normandy	201148	NA
Upper Normandy	201149	0
Upper Normandy	201150	0
Upper Normandy	201151	NA
Upper Normandy	201152	NA
Upper Normandy	201201	NA
Upper Normandy	201202	491.2619
Upper Normandy	201203	1065.278
Upper Normandy	201204	294.2857
Upper Normandy	201205	601.2857
Upper Normandy	201206	827.0371
Upper Normandy	201207	1310.296
Upper Normandy	201208	913.5519
Upper Normandy	201209	1899.781
Upper Normandy	201210	1514.667
Upper Normandy	201211	1096.744
Upper Normandy	201212	747.5111
Upper Normandy	201213	643.3334
Upper Normandy	201214	NA
Upper Normandy	201241	0
Upper Normandy	201242	0
Upper Normandy	201243	0
Upper Normandy	201244	0
Upper Normandy	201245	0

Upper Normandy	201246	154.3333
Upper Normandy	201247	257.1667
Upper Normandy	201248	340.9
Upper Normandy	201249	246.3
Upper Normandy	201250	598.1833
Upper Normandy	201251	1224.417
Upper Normandy	201252	630.8
Upper Normandy	201301	901.1084
Upper Normandy	201302	690.15
Upper Normandy	201303	877.5001
Upper Normandy	201304	1608.841
Upper Normandy	201305	1486.513
Upper Normandy	201306	2099.454
Upper Normandy	201307	2281.196
Upper Normandy	201308	1819.465
Upper Normandy	201309	1078.567
Upper Normandy	201310	886.0555
Upper Normandy	201311	420.2778
Upper Normandy	201312	595.1865
Upper Normandy	201313	566.4603
Upper Normandy	201314	316.9524
Ile-de-France	200341	216.2222
Ile-de-France	200342	294.9207
Ile-de-France	200343	336.9841
Ile-de-France	200344	227.7262
Ile-de-France	200345	509.0808
Ile-de-France	200346	841.2904
Ile-de-France	200347	1197.384
Ile-de-France	200348	1733.485
Ile-de-France	200349	2131.369
Ile-de-France	200350	1917.6
Ile-de-France	200351	1725.224
Ile-de-France	200352	1248.682
Ile-de-France	200401	456.7766
Ile-de-France	200402	641.6826
Ile-de-France	200403	236.6825
Ile-de-France	200404	95.72222
Ile-de-France	200405	262
Ile-de-France	200406	309
Ile-de-France	200407	311.5
Ile-de-France	200408	47.26667
Ile-de-France	200409	0
Ile-de-France	200410	0
Ile-de-France	200411	92
Ile-de-France	200412	282.5
Ile-de-France	200413	301.8333

Ile-de-France	200414	550
Ile-de-France	200441	24.68116
Ile-de-France	200442	26.71015
Ile-de-France	200443	24.30435
Ile-de-France	200444	0
Ile-de-France	200445	0
Ile-de-France	200446	0
Ile-de-France	200447	0
Ile-de-France	200448	165.3333
Ile-de-France	200449	219.1111
Ile-de-France	200450	376.2766
Ile-de-France	200451	347.9
Ile-de-France	200452	594.55
Ile-de-France	200453	862.0963
Ile-de-France	200501	773.0185
Ile-de-France	200502	905.1989
Ile-de-France	200503	902.5228
Ile-de-France	200504	1538.768
Ile-de-France	200505	2227.161
Ile-de-France	200506	2411.598
Ile-de-France	200507	1894.977
Ile-de-France	200508	945.0952
Ile-de-France	200509	851.7124
Ile-de-France	200510	683.5465
Ile-de-France	200511	810.3667
Ile-de-France	200512	723.1222
Ile-de-France	200513	585.4167
Ile-de-France	200514	570.3334
Ile-de-France	200541	0
Ile-de-France	200542	0
Ile-de-France	200543	0
Ile-de-France	200544	0
Ile-de-France	200545	0
Ile-de-France	200546	0
Ile-de-France	200547	0
Ile-de-France	200548	0
Ile-de-France	200549	61.84849
Ile-de-France	200550	129.9314
Ile-de-France	200551	147.7177
Ile-de-France	200552	226.9123
Ile-de-France	200601	171.3143
Ile-de-France	200602	416
Ile-de-France	200603	608.6053
Ile-de-France	200604	1042.137
Ile-de-France	200605	1054.124
Ile-de-France	200606	829.7219

Ile-de-France	200607	958.2369
Ile-de-France	200608	745.5588
Ile-de-France	200609	953.2543
Ile-de-France	200610	894.6581
Ile-de-France	200611	786.2807
Ile-de-France	200612	373.4226
Ile-de-France	200613	250.1367
Ile-de-France	200614	64.33334
Ile-de-France	200641	0
Ile-de-France	200642	139.8333
Ile-de-France	200643	148.75
Ile-de-France	200644	126.75
Ile-de-France	200645	0
Ile-de-France	200646	0
Ile-de-France	200647	53.06061
Ile-de-France	200648	130.1212
Ile-de-France	200649	350.1273
Ile-de-France	200650	398.4717
Ile-de-France	200651	617.4423
Ile-de-France	200652	830.517
Ile-de-France	200701	647.0128
Ile-de-France	200702	464.8747
Ile-de-France	200703	392.3181
Ile-de-France	200704	516.7917
Ile-de-France	200705	652.3799
Ile-de-France	200706	680.6989
Ile-de-France	200707	774.1133
Ile-de-France	200708	404.3464
Ile-de-France	200709	236.4927
Ile-de-France	200710	143.3394
Ile-de-France	200711	105.9487
Ile-de-France	200712	133.5385
Ile-de-France	200713	0
Ile-de-France	200714	NA
Ile-de-France	200741	0
Ile-de-France	200742	83
Ile-de-France	200743	204.6667
Ile-de-France	200744	143.3333
Ile-de-France	200745	117.9444
Ile-de-France	200746	45.88657
Ile-de-France	200747	60.5544
Ile-de-France	200748	45.37423
Ile-de-France	200749	146.5337
Ile-de-France	200750	309.6675
Ile-de-France	200751	477.673
Ile-de-France	200752	723.2678

Ile-de-France	200801	803.8884
Ile-de-France	200802	987.1364
Ile-de-France	200803	970.4973
Ile-de-France	200804	733.7343
Ile-de-France	200805	681.4589
Ile-de-France	200806	835.1957
Ile-de-France	200807	879.5309
Ile-de-France	200808	889.4689
Ile-de-France	200809	723.9927
Ile-de-France	200810	460.8864
Ile-de-France	200811	419.1752
Ile-de-France	200812	300.1514
Ile-de-France	200813	467.7249
Ile-de-France	200814	468.652
Ile-de-France	200841	35.24242
Ile-de-France	200842	72.02139
Ile-de-France	200843	134.3642
Ile-de-France	200844	151.1363
Ile-de-France	200845	177.0831
Ile-de-France	200846	132.4279
Ile-de-France	200847	195.6755
Ile-de-France	200848	326.1985
Ile-de-France	200849	532.8627
Ile-de-France	200850	897.0045
Ile-de-France	200851	1246.979
Ile-de-France	200852	1622.063
Ile-de-France	200901	1047.392
Ile-de-France	200902	1070.196
Ile-de-France	200903	1008.724
Ile-de-France	200904	1127.708
Ile-de-France	200905	670.7303
Ile-de-France	200906	513.4354
Ile-de-France	200907	396.0959
Ile-de-France	200908	283.2767
Ile-de-France	200909	344.0171
Ile-de-France	200910	247.4763
Ile-de-France	200911	331.0101
Ile-de-France	200912	383.5303
Ile-de-France	200913	373.6111
Ile-de-France	200914	360.9127
Ile-de-France	201041	0
Ile-de-France	201042	29.54546
Ile-de-France	201043	113.8182
Ile-de-France	201044	144.5455
Ile-de-France	201045	170.2121
Ile-de-France	201046	353.096

Ile-de-France	201047	456.1218
Ile-de-France	201048	748.5945
Ile-de-France	201049	1123.845
Ile-de-France	201050	1329.774
Ile-de-France	201051	1570.722
Ile-de-France	201052	1172.792
Ile-de-France	201101	1178.144
Ile-de-France	201102	1028.211
Ile-de-France	201103	947.3621
Ile-de-France	201104	697.3497
Ile-de-France	201105	715.2183
Ile-de-France	201106	534.051
Ile-de-France	201107	305.0985
Ile-de-France	201108	217.3224
Ile-de-France	201109	107.9141
Ile-de-France	201110	232.5933
Ile-de-France	201111	233.6074
Ile-de-France	201112	656.2592
Ile-de-France	201113	398.6111
Ile-de-France	201114	427.1111
Ile-de-France	201141	0
Ile-de-France	201142	0
Ile-de-France	201143	0
Ile-de-France	201144	63.13334
Ile-de-France	201145	91.73334
Ile-de-France	201146	118.7556
Ile-de-France	201147	60.65359
Ile-de-France	201148	87.70588
Ile-de-France	201149	166.7177
Ile-de-France	201150	290.8745
Ile-de-France	201151	289.7844
Ile-de-France	201152	220.1212
Ile-de-France	201201	188.0512
Ile-de-France	201202	265.463
Ile-de-France	201203	423.1579
Ile-de-France	201204	510.5116
Ile-de-France	201205	592.0504
Ile-de-France	201206	939.9319
Ile-de-France	201207	1387.616
Ile-de-France	201208	1433.846
Ile-de-France	201209	775.5511
Ile-de-France	201210	633.3172
Ile-de-France	201211	454.2127
Ile-de-France	201212	432.7646
Ile-de-France	201213	361.3333
Ile-de-France	201214	388.212

Ile-de-France	201241	0
Ile-de-France	201242	0
Ile-de-France	201243	102.6667
Ile-de-France	201244	218.8485
Ile-de-France	201245	266.0682
Ile-de-France	201246	210.9048
Ile-de-France	201247	244.1111
Ile-de-France	201248	422.9023
Ile-de-France	201249	758.5226
Ile-de-France	201250	1009.689
Ile-de-France	201251	1311.708
Ile-de-France	201252	1045.375
Ile-de-France	201301	852.0167
Ile-de-France	201302	1138.083
Ile-de-France	201303	1052.278
Ile-de-France	201304	1690.673
Ile-de-France	201305	1860.746
Ile-de-France	201306	1764.92
Ile-de-France	201307	1348.7
Ile-de-France	201308	1393.063
Ile-de-France	201309	1117.894
Ile-de-France	201310	773.5632
Ile-de-France	201311	519.5555
Ile-de-France	201312	406.3333
Ile-de-France	201313	429.1302
Ile-de-France	201314	335.7905
Lorraine	200341	82.90476
Lorraine	200342	91.76191
Lorraine	200343	81.85715
Lorraine	200344	71.09524
Lorraine	200345	159.0857
Lorraine	200346	325.7072
Lorraine	200347	662.7349
Lorraine	200348	1075.922
Lorraine	200349	1549.898
Lorraine	200350	1850.201
Lorraine	200351	1942.163
Lorraine	200352	1423.709
Lorraine	200401	1752.743
Lorraine	200402	770.4667
Lorraine	200403	621.3334
Lorraine	200404	82.04762
Lorraine	200405	83.80952
Lorraine	200406	NA
Lorraine	200407	NA
Lorraine	200408	NA

Lorraine	200409	NA
Lorraine	200410	0
Lorraine	200411	NA
Lorraine	200412	NA
Lorraine	200413	NA
Lorraine	200414	0
Lorraine	200441	0
Lorraine	200442	0
Lorraine	200443	0
Lorraine	200444	0
Lorraine	200445	0
Lorraine	200446	0
Lorraine	200447	0
Lorraine	200448	173.5556
Lorraine	200449	203.5556
Lorraine	200450	420.3333
Lorraine	200451	189.0476
Lorraine	200452	144.5714
Lorraine	200453	0
Lorraine	200501	350.8
Lorraine	200502	542.5455
Lorraine	200503	642.869
Lorraine	200504	777.8775
Lorraine	200505	1327.852
Lorraine	200506	2091.061
Lorraine	200507	1762.584
Lorraine	200508	1326.394
Lorraine	200509	703.1111
Lorraine	200510	1030.5
Lorraine	200511	645
Lorraine	200512	432.5139
Lorraine	200513	NA
Lorraine	200514	NA
Lorraine	200541	0
Lorraine	200542	0
Lorraine	200543	0
Lorraine	200544	0
Lorraine	200545	0
Lorraine	200546	0
Lorraine	200547	0
Lorraine	200548	0
Lorraine	200549	0
Lorraine	200550	0
Lorraine	200551	0
Lorraine	200552	0
Lorraine	200601	0

Lorraine	200602	135.5833
Lorraine	200603	219.6833
Lorraine	200604	562.6111
Lorraine	200605	558.494
Lorraine	200606	1004.672
Lorraine	200607	932.9545
Lorraine	200608	495.7273
Lorraine	200609	664.4697
Lorraine	200610	253.25
Lorraine	200611	278.25
Lorraine	200612	78.26667
Lorraine	200613	93.53334
Lorraine	200614	NA
Lorraine	200641	0
Lorraine	200642	0
Lorraine	200643	0
Lorraine	200644	0
Lorraine	200645	0
Lorraine	200646	0
Lorraine	200647	0
Lorraine	200648	0
Lorraine	200649	0
Lorraine	200650	0
Lorraine	200651	0
Lorraine	200652	0
Lorraine	200701	150.1818
Lorraine	200702	247.4546
Lorraine	200703	343.1516
Lorraine	200704	445.9813
Lorraine	200705	846.5028
Lorraine	200706	1223.408
Lorraine	200707	2035.872
Lorraine	200708	711.6889
Lorraine	200709	618.9555
Lorraine	200710	763.6333
Lorraine	200711	515.25
Lorraine	200712	NA
Lorraine	200713	NA
Lorraine	200714	NA
Lorraine	200741	0
Lorraine	200742	0
Lorraine	200743	0
Lorraine	200744	0
Lorraine	200745	92.13334
Lorraine	200746	101.6667
Lorraine	200747	201.5407

Lorraine	200748	288.6592
Lorraine	200749	253.3333
Lorraine	200750	308.5744
Lorraine	200751	318.9103
Lorraine	200752	633.25
Lorraine	200801	700.8571
Lorraine	200802	841.6905
Lorraine	200803	813.4095
Lorraine	200804	972.0389
Lorraine	200805	879.8778
Lorraine	200806	761.1686
Lorraine	200807	834.254
Lorraine	200808	837.2
Lorraine	200809	1204
Lorraine	200810	951.5852
Lorraine	200811	1204
Lorraine	200812	638.8889
Lorraine	200813	220.3333
Lorraine	200814	93.33334
Lorraine	200841	NA
Lorraine	200842	119
Lorraine	200843	89.91667
Lorraine	200844	88.41667
Lorraine	200845	0
Lorraine	200846	0
Lorraine	200847	0
Lorraine	200848	0
Lorraine	200849	103.5385
Lorraine	200850	318.3419
Lorraine	200851	699.9385
Lorraine	200852	796.1038
Lorraine	200901	1017.058
Lorraine	200902	1117.154
Lorraine	200903	1181.738
Lorraine	200904	1350.525
Lorraine	200905	964.4838
Lorraine	200906	807.8271
Lorraine	200907	497
Lorraine	200908	446.9333
Lorraine	200909	193.5556
Lorraine	200910	393.5555
Lorraine	200911	252.5333
Lorraine	200912	315.7333
Lorraine	200913	0
Lorraine	200914	NA
Lorraine	201041	0

Lorraine	201042	0
Lorraine	201043	0
Lorraine	201044	0
Lorraine	201045	0
Lorraine	201046	0
Lorraine	201047	55.76191
Lorraine	201048	182.4286
Lorraine	201049	209.037
Lorraine	201050	199.3386
Lorraine	201051	217.3333
Lorraine	201052	278.3333
Lorraine	201101	584.5609
Lorraine	201102	1054.975
Lorraine	201103	1296.597
Lorraine	201104	1361.001
Lorraine	201105	1687.692
Lorraine	201106	1553.114
Lorraine	201107	1178.204
Lorraine	201108	737.4896
Lorraine	201109	731.6
Lorraine	201110	354.6
Lorraine	201111	225.5556
Lorraine	201112	78.88889
Lorraine	201113	117
Lorraine	201114	281
Lorraine	201141	0
Lorraine	201142	0
Lorraine	201143	0
Lorraine	201144	0
Lorraine	201145	0
Lorraine	201146	NA
Lorraine	201147	NA
Lorraine	201148	NA
Lorraine	201149	144.3556
Lorraine	201150	468.6889
Lorraine	201151	292.0222
Lorraine	201152	220.6667
Lorraine	201201	36.27273
Lorraine	201202	82.60606
Lorraine	201203	157.2511
Lorraine	201204	304.6753
Lorraine	201205	468.3117
Lorraine	201206	830.631
Lorraine	201207	976.5796
Lorraine	201208	1196.604
Lorraine	201209	881.899

Lorraine	201210	788.5691
Lorraine	201211	591.7046
Lorraine	201212	449.9659
Lorraine	201213	563.6591
Lorraine	201214	619.7222
Lorraine	201241	0
Lorraine	201242	0
Lorraine	201243	0
Lorraine	201244	0
Lorraine	201245	0
Lorraine	201246	0
Lorraine	201247	0
Lorraine	201248	101.3571
Lorraine	201249	265.8333
Lorraine	201250	462.2167
Lorraine	201251	563.5757
Lorraine	201252	1101.148
Lorraine	201301	935.5403
Lorraine	201302	960.209
Lorraine	201303	987.2001
Lorraine	201304	1427.605
Lorraine	201305	2009.724
Lorraine	201306	1905.726
Lorraine	201307	1801.879
Lorraine	201308	1579.08
Lorraine	201309	1254.684
Lorraine	201310	734.0353
Lorraine	201311	430.8384
Lorraine	201312	218.6182
Lorraine	201313	152.4935
Lorraine	201314	150.1676
Nord-Pas-de-Calais	200341	NA
Nord-Pas-de-Calais	200342	NA
Nord-Pas-de-Calais	200343	824.1085
Nord-Pas-de-Calais	200344	1338.949
Nord-Pas-de-Calais	200345	1069.667
Nord-Pas-de-Calais	200346	1071.967
Nord-Pas-de-Calais	200347	1712.481
Nord-Pas-de-Calais	200348	1246.543
Nord-Pas-de-Calais	200349	1021.131
Nord-Pas-de-Calais	200350	1334.464
Nord-Pas-de-Calais	200351	869.181
Nord-Pas-de-Calais	200352	388.4
Nord-Pas-de-Calais	200401	0
Nord-Pas-de-Calais	200402	0
Nord-Pas-de-Calais	200403	0

Nord-Pas-de-Calais	200404	0
Nord-Pas-de-Calais	200405	NA
Nord-Pas-de-Calais	200406	NA
Nord-Pas-de-Calais	200407	NA
Nord-Pas-de-Calais	200408	NA
Nord-Pas-de-Calais	200409	NA
Nord-Pas-de-Calais	200410	NA
Nord-Pas-de-Calais	200411	NA
Nord-Pas-de-Calais	200412	NA
Nord-Pas-de-Calais	200413	NA
Nord-Pas-de-Calais	200414	NA
Nord-Pas-de-Calais	200441	NA
Nord-Pas-de-Calais	200442	NA
Nord-Pas-de-Calais	200443	NA
Nord-Pas-de-Calais	200444	NA
Nord-Pas-de-Calais	200445	NA
Nord-Pas-de-Calais	200446	NA
Nord-Pas-de-Calais	200447	NA
Nord-Pas-de-Calais	200448	NA
Nord-Pas-de-Calais	200449	294.6667
Nord-Pas-de-Calais	200450	420.6667
Nord-Pas-de-Calais	200451	0
Nord-Pas-de-Calais	200452	0
Nord-Pas-de-Calais	200453	120.75
Nord-Pas-de-Calais	200501	592.1667
Nord-Pas-de-Calais	200502	897.9167
Nord-Pas-de-Calais	200503	1205.729
Nord-Pas-de-Calais	200504	NA
Nord-Pas-de-Calais	200505	NA
Nord-Pas-de-Calais	200506	NA
Nord-Pas-de-Calais	200507	NA
Nord-Pas-de-Calais	200508	966.6191
Nord-Pas-de-Calais	200509	1413.5
Nord-Pas-de-Calais	200510	1042.5
Nord-Pas-de-Calais	200511	650
Nord-Pas-de-Calais	200512	0
Nord-Pas-de-Calais	200513	421.1111
Nord-Pas-de-Calais	200514	NA
Nord-Pas-de-Calais	200541	NA
Nord-Pas-de-Calais	200542	0
Nord-Pas-de-Calais	200543	NA
Nord-Pas-de-Calais	200544	NA
Nord-Pas-de-Calais	200545	NA
Nord-Pas-de-Calais	200546	0
Nord-Pas-de-Calais	200547	0
Nord-Pas-de-Calais	200548	0

Nord-Pas-de-Calais	200549	0
Nord-Pas-de-Calais	200550	0
Nord-Pas-de-Calais	200551	0
Nord-Pas-de-Calais	200552	NA
Nord-Pas-de-Calais	200601	NA
Nord-Pas-de-Calais	200602	NA
Nord-Pas-de-Calais	200603	340.1667
Nord-Pas-de-Calais	200604	541.8182
Nord-Pas-de-Calais	200605	501.8182
Nord-Pas-de-Calais	200606	530.2303
Nord-Pas-de-Calais	200607	541.8
Nord-Pas-de-Calais	200608	453.4
Nord-Pas-de-Calais	200609	218.6667
Nord-Pas-de-Calais	200610	346.4889
Nord-Pas-de-Calais	200611	648.6111
Nord-Pas-de-Calais	200612	1020.378
Nord-Pas-de-Calais	200613	1229.4
Nord-Pas-de-Calais	200614	NA
Nord-Pas-de-Calais	200641	NA
Nord-Pas-de-Calais	200642	NA
Nord-Pas-de-Calais	200643	239.3333
Nord-Pas-de-Calais	200644	230.3333
Nord-Pas-de-Calais	200645	217.8333
Nord-Pas-de-Calais	200646	0
Nord-Pas-de-Calais	200647	0
Nord-Pas-de-Calais	200648	257.3333
Nord-Pas-de-Calais	200649	576
Nord-Pas-de-Calais	200650	818
Nord-Pas-de-Calais	200651	566.6667
Nord-Pas-de-Calais	200652	503.1
Nord-Pas-de-Calais	200701	274.1259
Nord-Pas-de-Calais	200702	427.0984
Nord-Pas-de-Calais	200703	202.381
Nord-Pas-de-Calais	200704	295.0843
Nord-Pas-de-Calais	200705	510.5641
Nord-Pas-de-Calais	200706	1270.046
Nord-Pas-de-Calais	200707	1376.143
Nord-Pas-de-Calais	200708	1484.698
Nord-Pas-de-Calais	200709	858.3096
Nord-Pas-de-Calais	200710	549.5833
Nord-Pas-de-Calais	200711	NA
Nord-Pas-de-Calais	200712	NA
Nord-Pas-de-Calais	200713	NA
Nord-Pas-de-Calais	200714	NA
Nord-Pas-de-Calais	200741	NA
Nord-Pas-de-Calais	200742	NA

Nord-Pas-de-Calais	200743	NA
Nord-Pas-de-Calais	200744	NA
Nord-Pas-de-Calais	200745	0
Nord-Pas-de-Calais	200746	0
Nord-Pas-de-Calais	200747	0
Nord-Pas-de-Calais	200748	0
Nord-Pas-de-Calais	200749	0
Nord-Pas-de-Calais	200750	127.4167
Nord-Pas-de-Calais	200751	350.5833
Nord-Pas-de-Calais	200752	495.9167
Nord-Pas-de-Calais	200801	677.1429
Nord-Pas-de-Calais	200802	587.4762
Nord-Pas-de-Calais	200803	670.0715
Nord-Pas-de-Calais	200804	542.8666
Nord-Pas-de-Calais	200805	732.5555
Nord-Pas-de-Calais	200806	402.4445
Nord-Pas-de-Calais	200807	496.6667
Nord-Pas-de-Calais	200808	0
Nord-Pas-de-Calais	200809	83.66667
Nord-Pas-de-Calais	200810	260.4445
Nord-Pas-de-Calais	200811	562
Nord-Pas-de-Calais	200812	428.6667
Nord-Pas-de-Calais	200813	297.1111
Nord-Pas-de-Calais	200814	0
Nord-Pas-de-Calais	200841	0
Nord-Pas-de-Calais	200842	0
Nord-Pas-de-Calais	200843	NA
Nord-Pas-de-Calais	200844	NA
Nord-Pas-de-Calais	200845	NA
Nord-Pas-de-Calais	200846	0
Nord-Pas-de-Calais	200847	0
Nord-Pas-de-Calais	200848	0
Nord-Pas-de-Calais	200849	148.0833
Nord-Pas-de-Calais	200850	536.5454
Nord-Pas-de-Calais	200851	940.7606
Nord-Pas-de-Calais	200852	739.6182
Nord-Pas-de-Calais	200901	750.7482
Nord-Pas-de-Calais	200902	717.0222
Nord-Pas-de-Calais	200903	1243.988
Nord-Pas-de-Calais	200904	1093.693
Nord-Pas-de-Calais	200905	1163.411
Nord-Pas-de-Calais	200906	1351.952
Nord-Pas-de-Calais	200907	1188.667
Nord-Pas-de-Calais	200908	NA
Nord-Pas-de-Calais	200909	NA
Nord-Pas-de-Calais	200910	NA

Nord-Pas-de-Calais	200911	NA
Nord-Pas-de-Calais	200912	NA
Nord-Pas-de-Calais	200913	NA
Nord-Pas-de-Calais	200914	NA
Nord-Pas-de-Calais	201041	0
Nord-Pas-de-Calais	201042	0
Nord-Pas-de-Calais	201043	NA
Nord-Pas-de-Calais	201044	NA
Nord-Pas-de-Calais	201045	NA
Nord-Pas-de-Calais	201046	100.7619
Nord-Pas-de-Calais	201047	108.2857
Nord-Pas-de-Calais	201048	473.3333
Nord-Pas-de-Calais	201049	707.5079
Nord-Pas-de-Calais	201050	1190.667
Nord-Pas-de-Calais	201051	1274.325
Nord-Pas-de-Calais	201052	1361.349
Nord-Pas-de-Calais	201101	1320.906
Nord-Pas-de-Calais	201102	1251.795
Nord-Pas-de-Calais	201103	902.0941
Nord-Pas-de-Calais	201104	782.9829
Nord-Pas-de-Calais	201105	793.8485
Nord-Pas-de-Calais	201106	1020.855
Nord-Pas-de-Calais	201107	622.6
Nord-Pas-de-Calais	201108	629.8667
Nord-Pas-de-Calais	201109	432
Nord-Pas-de-Calais	201110	0
Nord-Pas-de-Calais	201111	NA
Nord-Pas-de-Calais	201112	NA
Nord-Pas-de-Calais	201113	NA
Nord-Pas-de-Calais	201114	NA
Nord-Pas-de-Calais	201141	NA
Nord-Pas-de-Calais	201142	0
Nord-Pas-de-Calais	201143	0
Nord-Pas-de-Calais	201144	0
Nord-Pas-de-Calais	201145	0
Nord-Pas-de-Calais	201146	0
Nord-Pas-de-Calais	201147	0
Nord-Pas-de-Calais	201148	0
Nord-Pas-de-Calais	201149	NA
Nord-Pas-de-Calais	201150	NA
Nord-Pas-de-Calais	201151	NA
Nord-Pas-de-Calais	201152	0
Nord-Pas-de-Calais	201201	0
Nord-Pas-de-Calais	201202	0
Nord-Pas-de-Calais	201203	193.0667
Nord-Pas-de-Calais	201204	352

Nord-Pas-de-Calais	201205	561.4857
Nord-Pas-de-Calais	201206	467.0397
Nord-Pas-de-Calais	201207	993.0184
Nord-Pas-de-Calais	201208	1419.827
Nord-Pas-de-Calais	201209	724.5043
Nord-Pas-de-Calais	201210	812.7778
Nord-Pas-de-Calais	201211	665.1666
Nord-Pas-de-Calais	201212	538.8445
Nord-Pas-de-Calais	201213	371.6
Nord-Pas-de-Calais	201214	185.0667
Nord-Pas-de-Calais	201241	NA
Nord-Pas-de-Calais	201242	NA
Nord-Pas-de-Calais	201243	NA
Nord-Pas-de-Calais	201244	NA
Nord-Pas-de-Calais	201245	0
Nord-Pas-de-Calais	201246	0
Nord-Pas-de-Calais	201247	0
Nord-Pas-de-Calais	201248	73.61111
Nord-Pas-de-Calais	201249	243.6667
Nord-Pas-de-Calais	201250	610.8334
Nord-Pas-de-Calais	201251	799.2315
Nord-Pas-de-Calais	201252	975.5926
Nord-Pas-de-Calais	201301	783.5768
Nord-Pas-de-Calais	201302	990.1905
Nord-Pas-de-Calais	201303	767.0688
Nord-Pas-de-Calais	201304	1080.577
Nord-Pas-de-Calais	201305	1245.084
Nord-Pas-de-Calais	201306	1486.566
Nord-Pas-de-Calais	201307	1946.707
Nord-Pas-de-Calais	201308	1640.741
Nord-Pas-de-Calais	201309	NA
Nord-Pas-de-Calais	201310	NA
Nord-Pas-de-Calais	201311	NA
Nord-Pas-de-Calais	201312	435.5556
Nord-Pas-de-Calais	201313	598.6667
Nord-Pas-de-Calais	201314	313.6667
Pays de la Loire	200341	102.8889
Pays de la Loire	200342	97.83334
Pays de la Loire	200343	184.1667
Pays de la Loire	200344	159.1667
Pays de la Loire	200345	521.2593
Pays de la Loire	200346	837.45
Pays de la Loire	200347	1606.8
Pays de la Loire	200348	2321.511
Pays de la Loire	200349	1932.056
Pays de la Loire	200350	2104

Pays de la Loire	200351	NA
Pays de la Loire	200352	NA
Pays de la Loire	200401	NA
Pays de la Loire	200402	1135
Pays de la Loire	200403	506.6667
Pays de la Loire	200404	226.1111
Pays de la Loire	200405	0
Pays de la Loire	200406	0
Pays de la Loire	200407	0
Pays de la Loire	200408	0
Pays de la Loire	200409	0
Pays de la Loire	200410	0
Pays de la Loire	200411	NA
Pays de la Loire	200412	NA
Pays de la Loire	200413	NA
Pays de la Loire	200414	NA
Pays de la Loire	200441	NA
Pays de la Loire	200442	NA
Pays de la Loire	200443	0
Pays de la Loire	200444	0
Pays de la Loire	200445	0
Pays de la Loire	200446	0
Pays de la Loire	200447	0
Pays de la Loire	200448	0
Pays de la Loire	200449	0
Pays de la Loire	200450	0
Pays de la Loire	200451	234.7778
Pays de la Loire	200452	358.4445
Pays de la Loire	200453	639.0477
Pays de la Loire	200501	550.3969
Pays de la Loire	200502	474.6032
Pays de la Loire	200503	813.3334
Pays de la Loire	200504	1057.778
Pays de la Loire	200505	1435.766
Pays de la Loire	200506	1517.832
Pays de la Loire	200507	1068.221
Pays de la Loire	200508	1253.593
Pays de la Loire	200509	1622.833
Pays de la Loire	200510	2270.53
Pays de la Loire	200511	2119.748
Pays de la Loire	200512	1571.621
Pays de la Loire	200513	538.2222
Pays de la Loire	200514	300.3333
Pays de la Loire	200541	NA
Pays de la Loire	200542	NA
Pays de la Loire	200543	NA

Pays de la Loire	200544	0
Pays de la Loire	200545	0
Pays de la Loire	200546	0
Pays de la Loire	200547	0
Pays de la Loire	200548	0
Pays de la Loire	200549	0
Pays de la Loire	200550	0
Pays de la Loire	200551	0
Pays de la Loire	200552	0
Pays de la Loire	200601	152.75
Pays de la Loire	200602	460.5
Pays de la Loire	200603	567
Pays de la Loire	200604	696.0909
Pays de la Loire	200605	561.5007
Pays de la Loire	200606	1096.585
Pays de la Loire	200607	835.0399
Pays de la Loire	200608	1086.465
Pays de la Loire	200609	640.4667
Pays de la Loire	200610	458.1905
Pays de la Loire	200611	479.7778
Pays de la Loire	200612	811.9048
Pays de la Loire	200613	967.2223
Pays de la Loire	200614	505.9445
Pays de la Loire	200641	0
Pays de la Loire	200642	0
Pays de la Loire	200643	197.5556
Pays de la Loire	200644	190.2222
Pays de la Loire	200645	182.5556
Pays de la Loire	200646	0
Pays de la Loire	200647	128.0667
Pays de la Loire	200648	124.0667
Pays de la Loire	200649	238.7429
Pays de la Loire	200650	161.8442
Pays de la Loire	200651	449.1429
Pays de la Loire	200652	495.6485
Pays de la Loire	200701	637.4222
Pays de la Loire	200702	675.4286
Pays de la Loire	200703	785.8062
Pays de la Loire	200704	1170.861
Pays de la Loire	200705	1638.782
Pays de la Loire	200706	1394.151
Pays de la Loire	200707	771.4662
Pays de la Loire	200708	617.6296
Pays de la Loire	200709	662.787
Pays de la Loire	200710	594.5278
Pays de la Loire	200711	583

Pays de la Loire	200712	349.4445
Pays de la Loire	200713	NA
Pays de la Loire	200714	NA
Pays de la Loire	200741	0
Pays de la Loire	200742	0
Pays de la Loire	200743	0
Pays de la Loire	200744	0
Pays de la Loire	200745	0
Pays de la Loire	200746	49.25926
Pays de la Loire	200747	68.25926
Pays de la Loire	200748	80.48148
Pays de la Loire	200749	119.7222
Pays de la Loire	200750	128.5
Pays de la Loire	200751	277.1111
Pays de la Loire	200752	204.0694
Pays de la Loire	200801	344.1083
Pays de la Loire	200802	367.6881
Pays de la Loire	200803	607.1143
Pays de la Loire	200804	866.1639
Pays de la Loire	200805	1017.406
Pays de la Loire	200806	1167.188
Pays de la Loire	200807	1245.932
Pays de la Loire	200808	1381.512
Pays de la Loire	200809	878.5
Pays de la Loire	200810	682.2728
Pays de la Loire	200811	502.415
Pays de la Loire	200812	395.125
Pays de la Loire	200813	477
Pays de la Loire	200814	395.5
Pays de la Loire	200841	0
Pays de la Loire	200842	0
Pays de la Loire	200843	0
Pays de la Loire	200844	117
Pays de la Loire	200845	128.6667
Pays de la Loire	200846	160.35
Pays de la Loire	200847	162.2833
Pays de la Loire	200848	261.5555
Pays de la Loire	200849	402.1861
Pays de la Loire	200850	737.4872
Pays de la Loire	200851	898.0865
Pays de la Loire	200852	1196.489
Pays de la Loire	200901	1462.409
Pays de la Loire	200902	2072.442
Pays de la Loire	200903	2426.287
Pays de la Loire	200904	1804.818
Pays de la Loire	200905	1510.677

Pays de la Loire	200906	1209.813
Pays de la Loire	200907	823.3883
Pays de la Loire	200908	620.3901
Pays de la Loire	200909	438.2487
Pays de la Loire	200910	375.873
Pays de la Loire	200911	748.9444
Pays de la Loire	200912	753.5
Pays de la Loire	200913	812
Pays de la Loire	200914	630.4166
Pays de la Loire	201041	0
Pays de la Loire	201042	0
Pays de la Loire	201043	0
Pays de la Loire	201044	194.8333
Pays de la Loire	201045	210.2143
Pays de la Loire	201046	228.2143
Pays de la Loire	201047	188.5368
Pays de la Loire	201048	321.2121
Pays de la Loire	201049	643.8278
Pays de la Loire	201050	978.7377
Pays de la Loire	201051	1033.151
Pays de la Loire	201052	1445.333
Pays de la Loire	201101	1253.252
Pays de la Loire	201102	1203.33
Pays de la Loire	201103	1161.064
Pays de la Loire	201104	1049.271
Pays de la Loire	201105	1302.938
Pays de la Loire	201106	1014.83
Pays de la Loire	201107	734.6178
Pays de la Loire	201108	501.1191
Pays de la Loire	201109	470.1984
Pays de la Loire	201110	340.2333
Pays de la Loire	201111	412.4666
Pays de la Loire	201112	347.3889
Pays de la Loire	201113	266.1111
Pays de la Loire	201114	99
Pays de la Loire	201141	0
Pays de la Loire	201142	0
Pays de la Loire	201143	0
Pays de la Loire	201144	0
Pays de la Loire	201145	0
Pays de la Loire	201146	0
Pays de la Loire	201147	0
Pays de la Loire	201148	0
Pays de la Loire	201149	0
Pays de la Loire	201150	0
Pays de la Loire	201151	774.3334

Pays de la Loire	201152	620
Pays de la Loire	201201	575.6667
Pays de la Loire	201202	0
Pays de la Loire	201203	113.2
Pays de la Loire	201204	187.5429
Pays de la Loire	201205	501.0346
Pays de la Loire	201206	714.8961
Pays de la Loire	201207	1170.703
Pays de la Loire	201208	1111.399
Pays de la Loire	201209	1321.327
Pays de la Loire	201210	1101.611
Pays de la Loire	201211	1262.222
Pays de la Loire	201212	1223.873
Pays de la Loire	201213	1002.75
Pays de la Loire	201214	844.2619
Pays de la Loire	201241	NA
Pays de la Loire	201242	NA
Pays de la Loire	201243	0
Pays de la Loire	201244	160.2222
Pays de la Loire	201245	203
Pays de la Loire	201246	426.6667
Pays de la Loire	201247	389.1667
Pays de la Loire	201248	733.5
Pays de la Loire	201249	967.4667
Pays de la Loire	201250	1593.533
Pays de la Loire	201251	1740.2
Pays de la Loire	201252	1064
Pays de la Loire	201301	1172.167
Pays de la Loire	201302	1013.389
Pays de la Loire	201303	981.9722
Pays de la Loire	201304	772.9471
Pays de la Loire	201305	1088.2
Pays de la Loire	201306	1736.536
Pays de la Loire	201307	2147.924
Pays de la Loire	201308	2126.05
Pays de la Loire	201309	2324.875
Pays de la Loire	201310	1682.292
Pays de la Loire	201311	911.8889
Pays de la Loire	201312	676.3889
Pays de la Loire	201313	573.2222
Pays de la Loire	201314	500.6667
Picardy	200341	NA
Picardy	200342	NA
Picardy	200343	1327.143
Picardy	200344	1363.333
Picardy	200345	765.7678

Picardy	200346	967.875
Picardy	200347	2576.293
Picardy	200348	2697.256
Picardy	200349	2919.443
Picardy	200350	1558.462
Picardy	200351	1516.5
Picardy	200352	NA
Picardy	200401	NA
Picardy	200402	NA
Picardy	200403	326.5
Picardy	200404	328.3333
Picardy	200405	0
Picardy	200406	NA
Picardy	200407	NA
Picardy	200408	NA
Picardy	200409	NA
Picardy	200410	NA
Picardy	200411	NA
Picardy	200412	NA
Picardy	200413	NA
Picardy	200414	NA
Picardy	200441	NA
Picardy	200442	NA
Picardy	200443	NA
Picardy	200444	NA
Picardy	200445	NA
Picardy	200446	NA
Picardy	200447	NA
Picardy	200448	0
Picardy	200449	0
Picardy	200450	113.9048
Picardy	200451	117.4286
Picardy	200452	273.9524
Picardy	200453	286
Picardy	200501	488.4028
Picardy	200502	523.5555
Picardy	200503	648.0833
Picardy	200504	997.8808
Picardy	200505	1142.797
Picardy	200506	1071.257
Picardy	200507	1325.821
Picardy	200508	1212.722
Picardy	200509	1675.106
Picardy	200510	1426.758
Picardy	200511	1741.994
Picardy	200512	1053.206

Picardy	200513	1230.556
Picardy	200514	NA
Picardy	200541	NA
Picardy	200542	NA
Picardy	200543	NA
Picardy	200544	NA
Picardy	200545	NA
Picardy	200546	NA
Picardy	200547	0
Picardy	200548	119.1905
Picardy	200549	128.7143
Picardy	200550	509.5238
Picardy	200551	305.3333
Picardy	200552	315.3333
Picardy	200601	0
Picardy	200602	503.8333
Picardy	200603	806.077
Picardy	200604	1362.303
Picardy	200605	1529.632
Picardy	200606	1581.04
Picardy	200607	1229.829
Picardy	200608	1254.629
Picardy	200609	589.9683
Picardy	200610	589.4445
Picardy	200611	334.8333
Picardy	200612	815
Picardy	200613	1346.5
Picardy	200614	921
Picardy	200641	NA
Picardy	200642	NA
Picardy	200643	0
Picardy	200644	0
Picardy	200645	0
Picardy	200646	93.76191
Picardy	200647	97.14286
Picardy	200648	91.57143
Picardy	200649	316.5714
Picardy	200650	NA
Picardy	200651	NA
Picardy	200652	NA
Picardy	200701	753.0556
Picardy	200702	777
Picardy	200703	885.1919
Picardy	200704	1455.606
Picardy	200705	2114.017
Picardy	200706	2248.976

Picardy	200707	1666.357
Picardy	200708	1188.583
Picardy	200709	662.75
Picardy	200710	618.2222
Picardy	200711	315.5556
Picardy	200712	NA
Picardy	200713	NA
Picardy	200714	NA
Picardy	200741	0
Picardy	200742	0
Picardy	200743	326.6667
Picardy	200744	280.6667
Picardy	200745	269.5
Picardy	200746	64.25926
Picardy	200747	67.44444
Picardy	200748	72.2963
Picardy	200749	77.33334
Picardy	200750	208.8593
Picardy	200751	662.137
Picardy	200752	964.6857
Picardy	200801	880.0063
Picardy	200802	1271.632
Picardy	200803	1654.702
Picardy	200804	1279
Picardy	200805	1231.714
Picardy	200806	989.8022
Picardy	200807	1121.272
Picardy	200808	537.8715
Picardy	200809	229.581
Picardy	200810	279.6095
Picardy	200811	354.2
Picardy	200812	363.8
Picardy	200813	547.5
Picardy	200814	594
Picardy	200841	0
Picardy	200842	NA
Picardy	200843	NA
Picardy	200844	NA
Picardy	200845	0
Picardy	200846	NA
Picardy	200847	NA
Picardy	200848	NA
Picardy	200849	631.7333
Picardy	200850	730.8889
Picardy	200851	1136.889
Picardy	200852	789.6296

Picardy	200901	138.3545
Picardy	200902	1075.498
Picardy	200903	1440.038
Picardy	200904	1265.429
Picardy	200905	932.8953
Picardy	200906	753.3334
Picardy	200907	378.2
Picardy	200908	491.4
Picardy	200909	296.5714
Picardy	200910	459.746
Picardy	200911	588.3889
Picardy	200912	599.25
Picardy	200913	386.75
Picardy	200914	482.75
Picardy	201041	87.11111
Picardy	201042	88.66666
Picardy	201043	71.66666
Picardy	201044	0
Picardy	201045	0
Picardy	201046	231.3333
Picardy	201047	441
Picardy	201048	1051.755
Picardy	201049	918.0667
Picardy	201050	1240.802
Picardy	201051	NA
Picardy	201052	NA
Picardy	201101	NA
Picardy	201102	1620.5
Picardy	201103	1289.937
Picardy	201104	1121.378
Picardy	201105	855.2889
Picardy	201106	728.3556
Picardy	201107	NA
Picardy	201108	NA
Picardy	201109	NA
Picardy	201110	0
Picardy	201111	0
Picardy	201112	NA
Picardy	201113	NA
Picardy	201114	NA
Picardy	201141	0
Picardy	201142	0
Picardy	201143	0
Picardy	201144	0
Picardy	201145	NA
Picardy	201146	NA

Picardy	201147	NA
Picardy	201148	0
Picardy	201149	0
Picardy	201150	NA
Picardy	201151	NA
Picardy	201152	NA
Picardy	201201	0
Picardy	201202	0
Picardy	201203	379.2381
Picardy	201204	547.7143
Picardy	201205	917.3207
Picardy	201206	971.4794
Picardy	201207	1399.927
Picardy	201208	1852.191
Picardy	201209	1187.2
Picardy	201210	907.9167
Picardy	201211	353.6945
Picardy	201212	713.3333
Picardy	201213	937.3334
Picardy	201214	NA
Picardy	201241	NA
Picardy	201242	NA
Picardy	201243	NA
Picardy	201244	NA
Picardy	201245	NA
Picardy	201246	NA
Picardy	201247	NA
Picardy	201248	NA
Picardy	201249	NA
Picardy	201250	1741.5
Picardy	201251	1686.111
Picardy	201252	1235.528
Picardy	201301	734.4445
Picardy	201302	907.5
Picardy	201303	935.9318
Picardy	201304	1582.943
Picardy	201305	2091.5
Picardy	201306	2280.667
Picardy	201307	2155.4
Picardy	201308	1334.4
Picardy	201309	1300.6
Picardy	201310	1616.3
Picardy	201311	819
Picardy	201312	645.3334
Picardy	201313	767.2778
Picardy	201314	1206.111

Provence-Alpes-Cote d'Azur	200341	0
Provence-Alpes-Cote d'Azur	200342	0
Provence-Alpes-Cote d'Azur	200343	0
Provence-Alpes-Cote d'Azur	200344	0
Provence-Alpes-Cote d'Azur	200345	260.4615
Provence-Alpes-Cote d'Azur	200346	714.8205
Provence-Alpes-Cote d'Azur	200347	1007.909
Provence-Alpes-Cote d'Azur	200348	1474.467
Provence-Alpes-Cote d'Azur	200349	1999.829
Provence-Alpes-Cote d'Azur	200350	1651.16
Provence-Alpes-Cote d'Azur	200351	1595.883
Provence-Alpes-Cote d'Azur	200352	363.6508
Provence-Alpes-Cote d'Azur	200401	890.7556
Provence-Alpes-Cote d'Azur	200402	288.9333
Provence-Alpes-Cote d'Azur	200403	260
Provence-Alpes-Cote d'Azur	200404	0
Provence-Alpes-Cote d'Azur	200405	0
Provence-Alpes-Cote d'Azur	200406	0
Provence-Alpes-Cote d'Azur	200407	144.4167
Provence-Alpes-Cote d'Azur	200408	207
Provence-Alpes-Cote d'Azur	200409	213.1667
Provence-Alpes-Cote d'Azur	200410	180
Provence-Alpes-Cote d'Azur	200411	167.3333
Provence-Alpes-Cote d'Azur	200412	NA
Provence-Alpes-Cote d'Azur	200413	NA
Provence-Alpes-Cote d'Azur	200414	NA
Provence-Alpes-Cote d'Azur	200441	0
Provence-Alpes-Cote d'Azur	200442	0
Provence-Alpes-Cote d'Azur	200443	0
Provence-Alpes-Cote d'Azur	200444	0
Provence-Alpes-Cote d'Azur	200445	0
Provence-Alpes-Cote d'Azur	200446	0
Provence-Alpes-Cote d'Azur	200447	0
Provence-Alpes-Cote d'Azur	200448	0
Provence-Alpes-Cote d'Azur	200449	98.4
Provence-Alpes-Cote d'Azur	200450	136.0667
Provence-Alpes-Cote d'Azur	200451	240.6268
Provence-Alpes-Cote d'Azur	200452	384.5732
Provence-Alpes-Cote d'Azur	200453	548.6436
Provence-Alpes-Cote d'Azur	200501	1075.083
Provence-Alpes-Cote d'Azur	200502	1238.273
Provence-Alpes-Cote d'Azur	200503	1631.325
Provence-Alpes-Cote d'Azur	200504	1821.222
Provence-Alpes-Cote d'Azur	200505	2337.183
Provence-Alpes-Cote d'Azur	200506	2169.235
Provence-Alpes-Cote d'Azur	200507	1203.013

Provence-Alpes-Cote d'Azur	200508	825.0596
Provence-Alpes-Cote d'Azur	200509	694.508
Provence-Alpes-Cote d'Azur	200510	554.1667
Provence-Alpes-Cote d'Azur	200511	458.25
Provence-Alpes-Cote d'Azur	200512	202.5
Provence-Alpes-Cote d'Azur	200513	107.25
Provence-Alpes-Cote d'Azur	200514	0
Provence-Alpes-Cote d'Azur	200541	0
Provence-Alpes-Cote d'Azur	200542	0
Provence-Alpes-Cote d'Azur	200543	0
Provence-Alpes-Cote d'Azur	200544	0
Provence-Alpes-Cote d'Azur	200545	0
Provence-Alpes-Cote d'Azur	200546	0
Provence-Alpes-Cote d'Azur	200547	0
Provence-Alpes-Cote d'Azur	200548	0
Provence-Alpes-Cote d'Azur	200549	0
Provence-Alpes-Cote d'Azur	200550	0
Provence-Alpes-Cote d'Azur	200551	0
Provence-Alpes-Cote d'Azur	200552	101.9111
Provence-Alpes-Cote d'Azur	200601	147.2496
Provence-Alpes-Cote d'Azur	200602	210.8257
Provence-Alpes-Cote d'Azur	200603	354.2393
Provence-Alpes-Cote d'Azur	200604	471.686
Provence-Alpes-Cote d'Azur	200605	618.5289
Provence-Alpes-Cote d'Azur	200606	557.2675
Provence-Alpes-Cote d'Azur	200607	277.5758
Provence-Alpes-Cote d'Azur	200608	221
Provence-Alpes-Cote d'Azur	200609	343.3571
Provence-Alpes-Cote d'Azur	200610	529.5178
Provence-Alpes-Cote d'Azur	200611	556.6115
Provence-Alpes-Cote d'Azur	200612	810.739
Provence-Alpes-Cote d'Azur	200613	425.9091
Provence-Alpes-Cote d'Azur	200614	213
Provence-Alpes-Cote d'Azur	200641	0
Provence-Alpes-Cote d'Azur	200642	0
Provence-Alpes-Cote d'Azur	200643	0
Provence-Alpes-Cote d'Azur	200644	0
Provence-Alpes-Cote d'Azur	200645	0
Provence-Alpes-Cote d'Azur	200646	0
Provence-Alpes-Cote d'Azur	200647	0
Provence-Alpes-Cote d'Azur	200648	0
Provence-Alpes-Cote d'Azur	200649	0
Provence-Alpes-Cote d'Azur	200650	92.11111
Provence-Alpes-Cote d'Azur	200651	384.2963
Provence-Alpes-Cote d'Azur	200652	680.291
Provence-Alpes-Cote d'Azur	200701	899.2361

Provence-Alpes-Cote d'Azur	200702	615.3965
Provence-Alpes-Cote d'Azur	200703	939.9359
Provence-Alpes-Cote d'Azur	200704	1451.91
Provence-Alpes-Cote d'Azur	200705	1759.138
Provence-Alpes-Cote d'Azur	200706	1456.07
Provence-Alpes-Cote d'Azur	200707	1151.018
Provence-Alpes-Cote d'Azur	200708	1118.845
Provence-Alpes-Cote d'Azur	200709	556.4854
Provence-Alpes-Cote d'Azur	200710	144.1111
Provence-Alpes-Cote d'Azur	200711	141.2222
Provence-Alpes-Cote d'Azur	200712	111.3333
Provence-Alpes-Cote d'Azur	200713	150.1111
Provence-Alpes-Cote d'Azur	200714	NA
Provence-Alpes-Cote d'Azur	200741	0
Provence-Alpes-Cote d'Azur	200742	0
Provence-Alpes-Cote d'Azur	200743	0
Provence-Alpes-Cote d'Azur	200744	0
Provence-Alpes-Cote d'Azur	200745	0
Provence-Alpes-Cote d'Azur	200746	0
Provence-Alpes-Cote d'Azur	200747	0
Provence-Alpes-Cote d'Azur	200748	71.9
Provence-Alpes-Cote d'Azur	200749	92.56667
Provence-Alpes-Cote d'Azur	200750	234.6606
Provence-Alpes-Cote d'Azur	200751	650.4546
Provence-Alpes-Cote d'Azur	200752	734.3636
Provence-Alpes-Cote d'Azur	200801	953.106
Provence-Alpes-Cote d'Azur	200802	794.3523
Provence-Alpes-Cote d'Azur	200803	807.5828
Provence-Alpes-Cote d'Azur	200804	1023.706
Provence-Alpes-Cote d'Azur	200805	1152.17
Provence-Alpes-Cote d'Azur	200806	818.1103
Provence-Alpes-Cote d'Azur	200807	941.625
Provence-Alpes-Cote d'Azur	200808	649.8149
Provence-Alpes-Cote d'Azur	200809	603
Provence-Alpes-Cote d'Azur	200810	252.1296
Provence-Alpes-Cote d'Azur	200811	222.1944
Provence-Alpes-Cote d'Azur	200812	223.7593
Provence-Alpes-Cote d'Azur	200813	269.4833
Provence-Alpes-Cote d'Azur	200814	214.35
Provence-Alpes-Cote d'Azur	200841	0
Provence-Alpes-Cote d'Azur	200842	0
Provence-Alpes-Cote d'Azur	200843	0
Provence-Alpes-Cote d'Azur	200844	0
Provence-Alpes-Cote d'Azur	200845	0
Provence-Alpes-Cote d'Azur	200846	0
Provence-Alpes-Cote d'Azur	200847	0

Provence-Alpes-Cote d'Azur	200848	83.77778
Provence-Alpes-Cote d'Azur	200849	158.9921
Provence-Alpes-Cote d'Azur	200850	351.1455
Provence-Alpes-Cote d'Azur	200851	415.5008
Provence-Alpes-Cote d'Azur	200852	811.9046
Provence-Alpes-Cote d'Azur	200901	1077.308
Provence-Alpes-Cote d'Azur	200902	1114.39
Provence-Alpes-Cote d'Azur	200903	1110.02
Provence-Alpes-Cote d'Azur	200904	1610.78
Provence-Alpes-Cote d'Azur	200905	1090.588
Provence-Alpes-Cote d'Azur	200906	734.8535
Provence-Alpes-Cote d'Azur	200907	463.5509
Provence-Alpes-Cote d'Azur	200908	551.6771
Provence-Alpes-Cote d'Azur	200909	586.9681
Provence-Alpes-Cote d'Azur	200910	348.1077
Provence-Alpes-Cote d'Azur	200911	277.6889
Provence-Alpes-Cote d'Azur	200912	187.4444
Provence-Alpes-Cote d'Azur	200913	220.5556
Provence-Alpes-Cote d'Azur	200914	0
Provence-Alpes-Cote d'Azur	201041	127.6
Provence-Alpes-Cote d'Azur	201042	127.0667
Provence-Alpes-Cote d'Azur	201043	134.0667
Provence-Alpes-Cote d'Azur	201044	0
Provence-Alpes-Cote d'Azur	201045	0
Provence-Alpes-Cote d'Azur	201046	0
Provence-Alpes-Cote d'Azur	201047	0
Provence-Alpes-Cote d'Azur	201048	67.63637
Provence-Alpes-Cote d'Azur	201049	400.9798
Provence-Alpes-Cote d'Azur	201050	474.0693
Provence-Alpes-Cote d'Azur	201051	945.2275
Provence-Alpes-Cote d'Azur	201052	1380.136
Provence-Alpes-Cote d'Azur	201101	1759.917
Provence-Alpes-Cote d'Azur	201102	1604.61
Provence-Alpes-Cote d'Azur	201103	2072.294
Provence-Alpes-Cote d'Azur	201104	2694.135
Provence-Alpes-Cote d'Azur	201105	2377.512
Provence-Alpes-Cote d'Azur	201106	2150.301
Provence-Alpes-Cote d'Azur	201107	1385.156
Provence-Alpes-Cote d'Azur	201108	937.3539
Provence-Alpes-Cote d'Azur	201109	529.5472
Provence-Alpes-Cote d'Azur	201110	215.5429
Provence-Alpes-Cote d'Azur	201111	54.47619
Provence-Alpes-Cote d'Azur	201112	87.26667
Provence-Alpes-Cote d'Azur	201113	85.53334
Provence-Alpes-Cote d'Azur	201114	79.26667
Provence-Alpes-Cote d'Azur	201141	0

Provence-Alpes-Cote d'Azur	201142	0
Provence-Alpes-Cote d'Azur	201143	0
Provence-Alpes-Cote d'Azur	201144	0
Provence-Alpes-Cote d'Azur	201145	0
Provence-Alpes-Cote d'Azur	201146	0
Provence-Alpes-Cote d'Azur	201147	0
Provence-Alpes-Cote d'Azur	201148	0
Provence-Alpes-Cote d'Azur	201149	0
Provence-Alpes-Cote d'Azur	201150	0
Provence-Alpes-Cote d'Azur	201151	0
Provence-Alpes-Cote d'Azur	201152	227.5
Provence-Alpes-Cote d'Azur	201201	438.5449
Provence-Alpes-Cote d'Azur	201202	794.7967
Provence-Alpes-Cote d'Azur	201203	1140.853
Provence-Alpes-Cote d'Azur	201204	1434.214
Provence-Alpes-Cote d'Azur	201205	2086.039
Provence-Alpes-Cote d'Azur	201206	1763.869
Provence-Alpes-Cote d'Azur	201207	2592.356
Provence-Alpes-Cote d'Azur	201208	2764.646
Provence-Alpes-Cote d'Azur	201209	1518.991
Provence-Alpes-Cote d'Azur	201210	771.2355
Provence-Alpes-Cote d'Azur	201211	289.5274
Provence-Alpes-Cote d'Azur	201212	156.0417
Provence-Alpes-Cote d'Azur	201213	48.95834
Provence-Alpes-Cote d'Azur	201214	112.1111
Provence-Alpes-Cote d'Azur	201241	0
Provence-Alpes-Cote d'Azur	201242	0
Provence-Alpes-Cote d'Azur	201243	0
Provence-Alpes-Cote d'Azur	201244	63.70834
Provence-Alpes-Cote d'Azur	201245	75.08334
Provence-Alpes-Cote d'Azur	201246	85.55093
Provence-Alpes-Cote d'Azur	201247	163.6445
Provence-Alpes-Cote d'Azur	201248	450.4042
Provence-Alpes-Cote d'Azur	201249	615.1048
Provence-Alpes-Cote d'Azur	201250	1083.565
Provence-Alpes-Cote d'Azur	201251	1755.972
Provence-Alpes-Cote d'Azur	201252	2249.19
Provence-Alpes-Cote d'Azur	201301	1832.087
Provence-Alpes-Cote d'Azur	201302	1861.957
Provence-Alpes-Cote d'Azur	201303	1951.655
Provence-Alpes-Cote d'Azur	201304	2206.261
Provence-Alpes-Cote d'Azur	201305	2409.598
Provence-Alpes-Cote d'Azur	201306	2398.415
Provence-Alpes-Cote d'Azur	201307	2014.356
Provence-Alpes-Cote d'Azur	201308	2015.638
Provence-Alpes-Cote d'Azur	201309	1451.37

Provence-Alpes-Cote d'Azur	201310	992.1524
Provence-Alpes-Cote d'Azur	201311	701.2286
Provence-Alpes-Cote d'Azur	201312	351.9445
Provence-Alpes-Cote d'Azur	201313	170.3778
Provence-Alpes-Cote d'Azur	201314	76.4
Rhone-Alpes	200341	49.14943
Rhone-Alpes	200342	161.3899
Rhone-Alpes	200343	147.13
Rhone-Alpes	200344	195.478
Rhone-Alpes	200345	334.2794
Rhone-Alpes	200346	548.932
Rhone-Alpes	200347	999.2386
Rhone-Alpes	200348	1404.207
Rhone-Alpes	200349	1671.614
Rhone-Alpes	200350	2209.16
Rhone-Alpes	200351	1966.853
Rhone-Alpes	200352	1464.41
Rhone-Alpes	200401	831.498
Rhone-Alpes	200402	514.0072
Rhone-Alpes	200403	178.582
Rhone-Alpes	200404	138.8142
Rhone-Alpes	200405	75.74118
Rhone-Alpes	200406	116.3222
Rhone-Alpes	200407	73.92593
Rhone-Alpes	200408	71.48148
Rhone-Alpes	200409	0
Rhone-Alpes	200410	66.20834
Rhone-Alpes	200411	159.0556
Rhone-Alpes	200412	137.1806
Rhone-Alpes	200413	80.16666
Rhone-Alpes	200414	0
Rhone-Alpes	200441	0
Rhone-Alpes	200442	0
Rhone-Alpes	200443	0
Rhone-Alpes	200444	0
Rhone-Alpes	200445	0
Rhone-Alpes	200446	18.79365
Rhone-Alpes	200447	24.77778
Rhone-Alpes	200448	25.19048
Rhone-Alpes	200449	0
Rhone-Alpes	200450	0
Rhone-Alpes	200451	44.5
Rhone-Alpes	200452	165.75
Rhone-Alpes	200453	289.3
Rhone-Alpes	200501	501.2159
Rhone-Alpes	200502	610.8243

Rhone-Alpes	200503	978.6132
Rhone-Alpes	200504	1276.287
Rhone-Alpes	200505	1845.018
Rhone-Alpes	200506	2200.573
Rhone-Alpes	200507	1603.888
Rhone-Alpes	200508	1385.928
Rhone-Alpes	200509	1023.392
Rhone-Alpes	200510	873.7953
Rhone-Alpes	200511	691.363
Rhone-Alpes	200512	474.8504
Rhone-Alpes	200513	328.334
Rhone-Alpes	200514	291.0628
Rhone-Alpes	200541	0
Rhone-Alpes	200542	0
Rhone-Alpes	200543	0
Rhone-Alpes	200544	0
Rhone-Alpes	200545	0
Rhone-Alpes	200546	0
Rhone-Alpes	200547	0
Rhone-Alpes	200548	0
Rhone-Alpes	200549	0
Rhone-Alpes	200550	0
Rhone-Alpes	200551	0
Rhone-Alpes	200552	45.30952
Rhone-Alpes	200601	68.56571
Rhone-Alpes	200602	155.3626
Rhone-Alpes	200603	270.7036
Rhone-Alpes	200604	377.0088
Rhone-Alpes	200605	554.8231
Rhone-Alpes	200606	753.5302
Rhone-Alpes	200607	1288.483
Rhone-Alpes	200608	1062.939
Rhone-Alpes	200609	798.2757
Rhone-Alpes	200610	567.0797
Rhone-Alpes	200611	538.4614
Rhone-Alpes	200612	505.8459
Rhone-Alpes	200613	384.0313
Rhone-Alpes	200614	448.1645
Rhone-Alpes	200641	0
Rhone-Alpes	200642	0
Rhone-Alpes	200643	0
Rhone-Alpes	200644	0
Rhone-Alpes	200645	0
Rhone-Alpes	200646	0
Rhone-Alpes	200647	0
Rhone-Alpes	200648	0

Rhone-Alpes	200649	64.53334
Rhone-Alpes	200650	183.8842
Rhone-Alpes	200651	377.8601
Rhone-Alpes	200652	601.054
Rhone-Alpes	200701	576.0935
Rhone-Alpes	200702	751.6447
Rhone-Alpes	200703	663.246
Rhone-Alpes	200704	851.5888
Rhone-Alpes	200705	1097.95
Rhone-Alpes	200706	1341.69
Rhone-Alpes	200707	967.8647
Rhone-Alpes	200708	760.9412
Rhone-Alpes	200709	375.3623
Rhone-Alpes	200710	244.2085
Rhone-Alpes	200711	132.0332
Rhone-Alpes	200712	22.68628
Rhone-Alpes	200713	103
Rhone-Alpes	200714	NA
Rhone-Alpes	200741	NA
Rhone-Alpes	200742	0
Rhone-Alpes	200743	0
Rhone-Alpes	200744	0
Rhone-Alpes	200745	0
Rhone-Alpes	200746	0
Rhone-Alpes	200747	0
Rhone-Alpes	200748	47.58333
Rhone-Alpes	200749	137.2668
Rhone-Alpes	200750	311.4315
Rhone-Alpes	200751	399.3962
Rhone-Alpes	200752	438.9611
Rhone-Alpes	200801	580.8611
Rhone-Alpes	200802	884.6328
Rhone-Alpes	200803	1026.701
Rhone-Alpes	200804	1115.597
Rhone-Alpes	200805	1050.684
Rhone-Alpes	200806	1169.415
Rhone-Alpes	200807	1102.099
Rhone-Alpes	200808	942.2784
Rhone-Alpes	200809	884.1732
Rhone-Alpes	200810	638.7928
Rhone-Alpes	200811	528.9791
Rhone-Alpes	200812	440.4842
Rhone-Alpes	200813	503.2711
Rhone-Alpes	200814	397.3026
Rhone-Alpes	200841	0
Rhone-Alpes	200842	0

Rhone-Alpes	200843	0
Rhone-Alpes	200844	0
Rhone-Alpes	200845	0
Rhone-Alpes	200846	0
Rhone-Alpes	200847	27.18518
Rhone-Alpes	200848	33.72839
Rhone-Alpes	200849	81.84046
Rhone-Alpes	200850	167.2331
Rhone-Alpes	200851	442.8937
Rhone-Alpes	200852	566.1439
Rhone-Alpes	200901	852.6479
Rhone-Alpes	200902	1191.266
Rhone-Alpes	200903	1466.145
Rhone-Alpes	200904	1862.037
Rhone-Alpes	200905	1596.435
Rhone-Alpes	200906	1118.809
Rhone-Alpes	200907	783.75
Rhone-Alpes	200908	398.7781
Rhone-Alpes	200909	354.5502
Rhone-Alpes	200910	364.497
Rhone-Alpes	200911	268.3302
Rhone-Alpes	200912	201.6667
Rhone-Alpes	200913	105.5833
Rhone-Alpes	200914	90.66667
Rhone-Alpes	201041	0
Rhone-Alpes	201042	0
Rhone-Alpes	201043	0
Rhone-Alpes	201044	0
Rhone-Alpes	201045	16.87037
Rhone-Alpes	201046	21.59259
Rhone-Alpes	201047	23.03704
Rhone-Alpes	201048	53.8
Rhone-Alpes	201049	152.88
Rhone-Alpes	201050	363.3319
Rhone-Alpes	201051	588.8697
Rhone-Alpes	201052	856.388
Rhone-Alpes	201101	1315.212
Rhone-Alpes	201102	1273.451
Rhone-Alpes	201103	1363.8
Rhone-Alpes	201104	1578.769
Rhone-Alpes	201105	1935.826
Rhone-Alpes	201106	1921.912
Rhone-Alpes	201107	1660.552
Rhone-Alpes	201108	1070.417
Rhone-Alpes	201109	504.2619
Rhone-Alpes	201110	324.8943

Rhone-Alpes	201111	181.9125
Rhone-Alpes	201112	68.79166
Rhone-Alpes	201113	34.8
Rhone-Alpes	201114	0
Rhone-Alpes	201141	24.57407
Rhone-Alpes	201142	23.07407
Rhone-Alpes	201143	0
Rhone-Alpes	201144	0
Rhone-Alpes	201145	0
Rhone-Alpes	201146	0
Rhone-Alpes	201147	0
Rhone-Alpes	201148	0
Rhone-Alpes	201149	0
Rhone-Alpes	201150	0
Rhone-Alpes	201151	0
Rhone-Alpes	201152	60.7037
Rhone-Alpes	201201	245.5896
Rhone-Alpes	201202	340.6547
Rhone-Alpes	201203	554.1437
Rhone-Alpes	201204	671.0828
Rhone-Alpes	201205	1032.55
Rhone-Alpes	201206	1512.676
Rhone-Alpes	201207	1454.519
Rhone-Alpes	201208	1466.794
Rhone-Alpes	201209	1179.736
Rhone-Alpes	201210	1047.395
Rhone-Alpes	201211	888.9077
Rhone-Alpes	201212	645.4519
Rhone-Alpes	201213	503.4386
Rhone-Alpes	201214	370.8684
Rhone-Alpes	201241	0
Rhone-Alpes	201242	0
Rhone-Alpes	201243	0
Rhone-Alpes	201244	26.71795
Rhone-Alpes	201245	81.73219
Rhone-Alpes	201246	72.76923
Rhone-Alpes	201247	62.86222
Rhone-Alpes	201248	106.1427
Rhone-Alpes	201249	287.6412
Rhone-Alpes	201250	575.7064
Rhone-Alpes	201251	852.9135
Rhone-Alpes	201252	853.3449
Rhone-Alpes	201301	1081.405
Rhone-Alpes	201302	1270.218
Rhone-Alpes	201303	1521.149
Rhone-Alpes	201304	2117.586

Rhone-Alpes	201305	2704.858
Rhone-Alpes	201306	2476.358
Rhone-Alpes	201307	2027.843
Rhone-Alpes	201308	1513.762
Rhone-Alpes	201309	1075.817
Rhone-Alpes	201310	749.0636
Rhone-Alpes	201311	418.7233
Rhone-Alpes	201312	299.0988
Rhone-Alpes	201313	224.7269
Rhone-Alpes	201314	147.1149
